# Exploring the impact of the stargazin V143L mutation on the dynamics of the AMPA receptor: stargazin complex

**DOI:** 10.3389/fncel.2024.1505846

**Published:** 2025-01-17

**Authors:** Raquel P. Gouveia, Carlos A. V. Barreto, Rita Melo, Ana Luísa Carvalho, Irina S. Moreira

**Affiliations:** ^1^Department of Life Sciences, University of Coimbra, Calçada Martim de Freitas, Coimbra, Portugal; ^2^CNC—Center for Neuroscience and Cell Biology, CIBB—Center for Innovative Biomedicine and Biotechnology, University of Coimbra, Coimbra, Portugal; ^3^IIIs—Institute for Interdisciplinary Research, University of Coimbra, Coimbra, Portugal; ^4^Centro de Ciências e Tecnologias Nucleares and Departamento de Engenharia e Ciências Nucleares, Instituto Superior Técnico, Universidade de Lisboa, CTN, Bobadela LRS, Portugal

**Keywords:** stargazin, AMPAR, V143L mutation, intellectual disability, protein-protein interface, structure and dynamic

## Abstract

Stargazin, a transmembrane AMPAR regulatory protein (TARP), plays a crucial role in facilitating the transport of AMPA receptors to the cell surface, stabilising their localisation at synapses and influencing their gating properties. The primary objective of this study was to investigate the effect of the V143L mutation in stargazin, previously linked to intellectual disability, on the interaction between stargazin and AMPA receptors. To achieve this, we conducted a thorough examination of eight distinct molecular dynamics simulations of AMPA receptor-stargazin complexes, each associated with different conductance levels. Through extensive analysis of complex interface structures and dynamics, we revealed that the stargazin V143L mutation had a more pronounced destabilising effect on complexes with lower conductance levels than on the conductive states of the receptor, suggesting a potential association with impaired synaptic transmission in individuals with this mutation.

## Graphical abstract

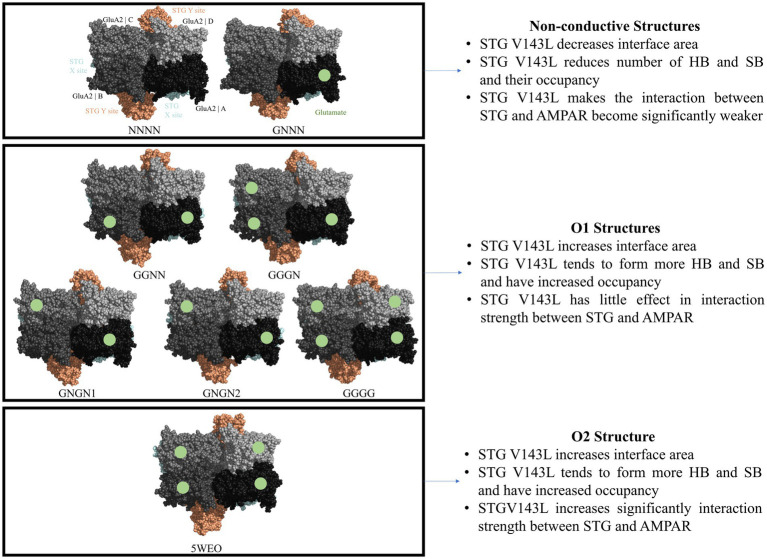

## 1 Introduction

**Figure 1:**
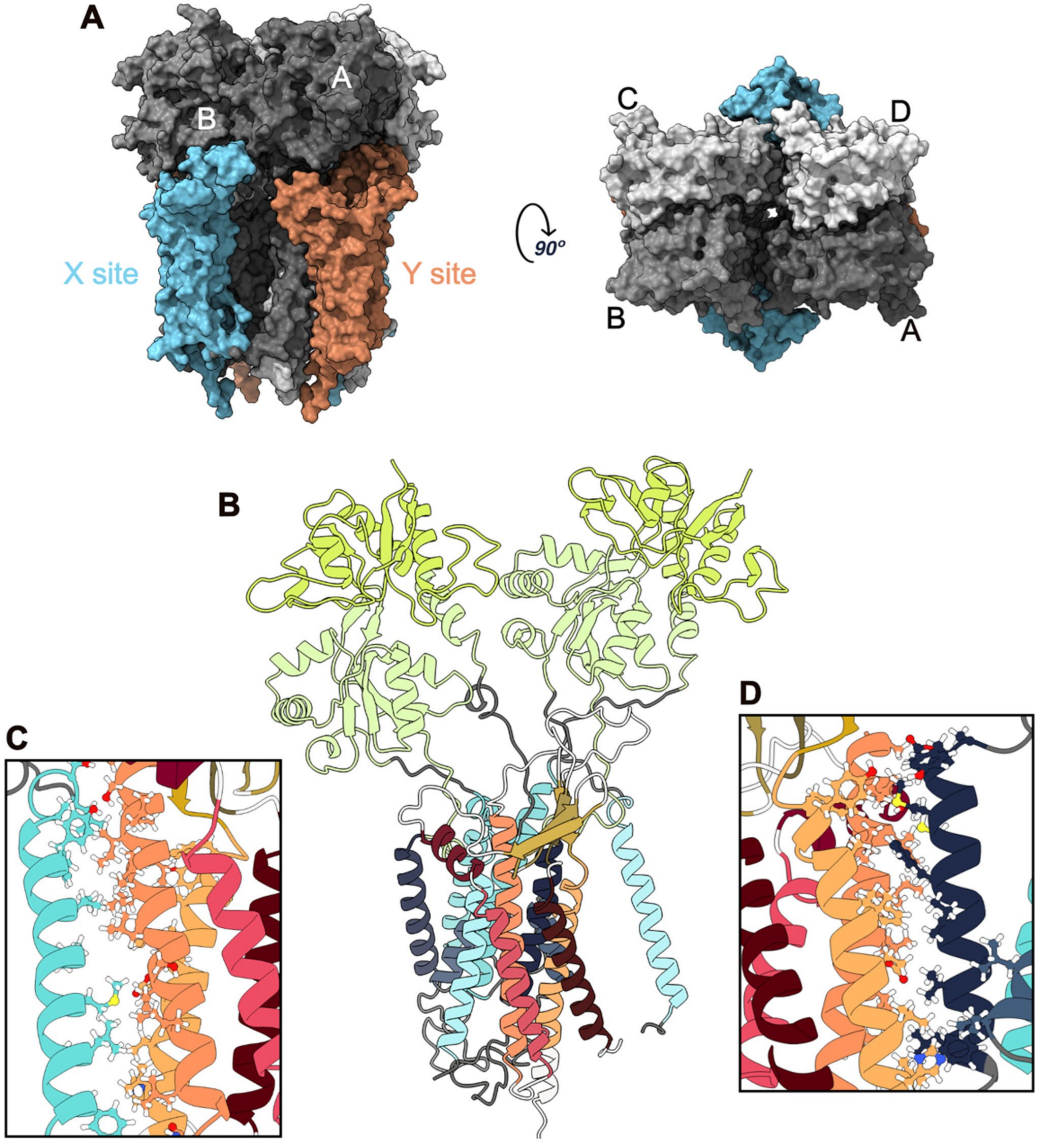
GluA2:STG Complex Structure. **(A)** Surface representation of the complex: side (left) and top (right) views. The ATD is not shown as it was removed from the MD simulations. Four GluA2 subunits are shown in different gray shades. The four STG bounded to GluA2 are coloured according to the site: blue for the X site and orange for the Y site. **(B)** Cartoon representation of an X-site with two GluA2 subunits (Main and Secondary) and the STG. M1-4 are colored in blue, and LBD-D1 and LBD-D2 are coloured in green. STG helices are coloured red-orange, and β-sheets are coloured yellow. **(C)** Interface between TMD3 of STG and M4 of Secondary GluA2. **(D)** Interface between M1 of Main GluA2 and TMD4 of the STG.

Glutamate is a key excitatory neurotransmitter in the Central Nervous System (CNS), and glutamatergic synapses are critical for the function of neuronal circuits underlying sensory and cognitive processes (Hansen et al., 2021). α-Amino-3-hydroxy-5-Methyl-4-isoxazole Propionic Acid Receptors (AMPARs) are ionotropic glutamate receptors that mediate rapid excitatory neurotransmission. The kinetics of these receptors are determined by their composition in four subunits (GluA1–GluA4) that assemble in homomeric or heteromeric tetramers and by the binding of auxiliary proteins (Hansen et al., 2021). The AMPAR composition depends on the brain region, cell type, and developmental stage. GluA1, GluA2, and GluA3 are predominantly expressed in the cortex, hippocampus, olfactory region, basal ganglia, lateral septum, and amygdala, whereas GluA4 is expressed more frequently in the cerebellum and reticular thalamic nuclei (Santos et al., 2009). These four subunits assemble into a Y-shaped structure consisting of three layers: Amino-Terminal Domain (ATD), Ligand-Binding Domain (LBD), and TransMembrane Domain (TMD). The TMD comprises four helices (M1-M4), of which three are transmembrane (M1, M3, and M4), and M2 is a re-entrant loop that forms the pore of the receptor channel (Greger et al., 2017). AMPAR have a specific arrangement of the four subunits across the three layers, forming a tetrameric assembly that displays two-fold rotational symmetry and exhibits high conformational flexibility (Twomey et al., 2019). Two dimers composed of A/B and C/D subunit pairs are present in the ATD layer. However, while the cross-dimer interface in this layer is between the B and D subunits, in the LBD it changes into the A and C subunits, connecting the A/D and B/C dimers (Figure 1A).

Several AMPAR regulatory proteins have also been identified. Among them, stargazin (STG), a Transmembrane AMPAR Regulatory Protein (TARP), is encoded by the Calcium voltage-gated Channel Secondary subunit Gamma 2 (*CACNG2*) gene and plays a major role in the regulation of AMPAR function. It is engaged in receptor trafficking to the cell surface and synaptic stabilisation, as well as in the modulation of receptor-gating properties (deactivation and desensitisation; Tomita et al., 2005; Twomey et al., 1979; Ben-Yaacov et al., 2017; Shaikh et al., 2016). STG is formed by a bundle of four helices that constitute the TMDs and two Extracellular Domains (ECDs). The first ECD is between TM1 and TM2, and is composed of four β-sheets (β1–β4) and a small ExtraCellular Helix (ECH) located immediately before TM2; the second ECD is between TM3 and TM4, consisting of one β-sheet (β5; Twomey et al., 2019; Twomey et al., 1979; Ben-Yaacov et al., 2017; Shaikh et al., 2016; Price et al., 2005; Vandenberghe et al., 2005).

In line with the structures elucidated to date, TMDs have been reported to be the main interacting domains between AMPAR and TARPs. M1, M2, and M4 from AMPAR are involved in the interactions with TM3 and TM4 from TARPs (Figure 1). Owing to its pseudo-fourfold symmetric structure, each AMPAR complex can interact with a maximum of four TARPs. The four available TMDs interfaces can be split into two groups: X TARP sites (common interfaces with AMPAR subunits A and B or C and D) and Y TARP sites (involving subunits A and D or B and C; Twomey et al., 2019). Different LDB interfaces are also involved in TARP interactions based on the site occupied (X or Y), possibly resulting in different biological outcomes (Twomey et al., 2019). Although the interaction at the X-site appears to modulate AMPAR gating kinetics (Dawe et al., 2016), the interaction at the Y-site, which implicates the flip/flop splice cassette, may affect AMPAR dynamics and regulate the rate of the channel-closing process and its desensitisation in the millisecond time region (Dawe et al., 2016; Milstein and Nicoll, 2008; Kott et al., 2007; Tomita et al., 2006). AMPAR-TARP assembly is also dependent on TARP, namely, its C-TERMinal domain (CTERM). Changes in the TARP ECD loops can also lead to the modulation of distinct AMPAR ECD sites, particularly the proximity between the TARP ECD and AMPAR LBD (Twomey et al., 2019). This fact, along with the ability of TARPs to interact with M1 and M2 from one AMPAR TMD subunit and M4 from another subunit, makes the assembly of AMPAR: TARPs almost unique.

When activated, AMPARs move from their baseline level (C) and reach up to four conductance levels (O1–O4; Yelshanskaya et al., 2022). Multiple conductance levels are believed to arise from different numbers of agonist molecules bound to receptor subunit LBDs. Recently, solved structures of AMPAR with STG with different occupancies of the LBD by the agonist glutamate have been published (Yelshanskaya et al., 2022). In this study, it was found that a minimum of two subunits must be bound to an agonist for the complex to open at the lowest conducting level O1. Furthermore, Yelshanskaya et al. have also shown that even when all four GluA2 AMPAR LBD subunits are bound to glutamate, channel conductance may not reach its maximal level (O4; Yelshanskaya et al., 2022), and that Glu can only bind to the LBDs of AMPAR subunits B and D after they are already bound to the same number of LBDs in GluA2 subunits A and C, indicating a non-equivalent contribution of receptor subunits to AMPAR gating.

Impaired excitatory transmission and plasticity have been implicated in neuropsychiatric disorders (Purcell et al., 2014; Yizhar et al., 2011). The *CACNG2* gene encoding stargazin is highly constrained and is considered an autism spectrum disorder candidate gene, intolerant to loss-of-function variants [(Rolland et al., 2023); SFARI database (Abrahams et al., 2013)]. It is also a candidate gene for neurodevelopmental disorders, based on the Genetrek database (Leblond et al., 2021). A *de novo* missense variant of STG (V143L) was identified in a patient presenting with non-syndromic intellectual disability (Hamdan et al., 2011). We have previously shown that this stargazin variant results in reduced binding to AMPAR subunits, and knock-in mice with the V143L variant displayed cognitive and social deficits, as well as hippocampal synaptic transmission defects (Caldeira et al., 2022). However, the exact effect of the V143L mutation on the structure and function of STG, and therefore on the AMPAR-STG complex, as well as its relationship with different AMPAR (GluA2 tetramer) conducting levels, remains unclear. In this study, we employed Molecular Dynamics (MD) simulations to explore the atomic-level intricacies of the AMPAR:STG interface across various conductance levels in complexes, including wild-type (WT) or V143L variants. Our analysis scrutinised the structural and dynamic disparities between WT STG and V143L containing complexes, shedding light on the nuanced effects of this mutation on the behaviour of the system. By meticulously dissecting these distinctions, we aimed to deepen our understanding of the molecular implications of V143L STG mutation and its impact on functional outcomes.

## 2 Materials and methods

### 2.1 Model construction

Following the structure reported by Yelshanskaya et al. (2022), we considered two types of GluA2 AMPAR subunits: Glu-bound (G) and Glu-unbonded (N) subunits. The three types of GluA2 dimers represent all possible combinations of the N and G monomers: GG, GN (equal to NG), and NN. The NN and GG dimers exhibit two-fold rotational symmetry, whereas the GN dimers are asymmetrical. The initial atomic models for the eight systems (NNNN, GNNN, GNGN1, GNGN2, GGNN, GGGN, GGGG, and 5WEO) were obtained from the Protein Data Bank (PDB) under the following IDs: PDB-IDs:7TNJ (Yelshanskaya et al., 2022), 7TNK (Yelshanskaya et al., 2022), 7TNL (Yelshanskaya et al., 2022), 7TNM (Yelshanskaya et al., 2022), 7TNN (Yelshanskaya et al., 2022), 7TNO (Yelshanskaya et al., 2022), 7TNP (Yelshanskaya et al., 2022), and 5WEO (Twomey et al., 2017). The GNGN arrangement consists of two structures with distinct conformations (GNGN1 and GNGN2). Complete three-dimensional (3D) structures of AMPAR-STG chimeras were obtained using the MODELLER package (Sali and Blundell, 1993), with which five models were constructed and later evaluated using the DOPE score, z-score (Wiederstein and Sippl, 2007; Sippl, 1993), LGscore (Wallner and Elofsson, 2003), and MaxSub (Wallner and Elofsson, 2003). The positive allosteric modulator CycloThiaZide (CTZ) and endogenous ligand glutamate (Glu) were preserved in their original positions. These were parameterised using the CHARMM General Force Field (Vanommeslaeghe et al., 2010; Yu et al., 2012). Mutated forms of STG (V143L) were constructed using PyMOL (Schrödinger, n.d.) by mutating the original valine residue to leucine.

### 2.2 Molecular dynamics simulations

MD simulations of STG Wild-Type (WT) and V143L mutated systems were performed using GROMACS 2018.4 (Abraham et al., 2018; Bekker et al., 1993) and CHARMM36 force field (Huang and Mackerell, 2013). The orientation of the complex in the membrane was determined using the oriented crystal of the AMPAR-STG chimera [PDB-ID:5WEO (Twomey et al., 2017)]. The systems were constructed using a CHARMM-GUI (Wu et al., 2014; Jo et al., 2008; Lee et al., 2018) membrane builder with a bilayer membrane of POPC:Cholesterol (9:1 ratio) to replicate the physiological environment. These simulation boxes were also hydrated using the TIP3 model of water and 0.15 M NaCl. The systems were subjected to initial minimisation using the steepest-descent algorithm for 50,000 steps. A Berendsen thermostat was used to increase the temperature of the system to 310 K, and a semi-isotropic pressure-coupling algorithm was used to increase the pressure to 1 bar. Simultaneously, the constraint forces of lipids and proteins successively decreased. Production runs were performed using the Nose-Hoover thermostat with a time constant of 1 ps and a semi-isotropic Parrinello–Rahman barostat with a time constant of 5 ps and compressibility of 4.5 × 10–5 bar-1. Electrostatic interactions were performed using a fast smooth Particle-Mesh Ewald (PME) with a cut-off of 12 Å. The H-bonds were constrained using a linear constraint solver. For each system, an initial run of 50 ns was performed to complete the equilibration of the systems, followed by three independent replicas of short-production MD simulations (50 ns each) to minimise changes in the initial conformation of the system. The rationale behind this approach was to prevent significant alterations to the system, while still gaining valuable insights. Additionally, the use of MM-PBSA has been demonstrated to yield more reliable energy values with shorter simulation times (Genheden and Ryde, 2015).

### 2.3 Analysis

Several computational analyses were applied to the simulations to study the interaction between STG and AMPAR and the differences between the mutated and WT systems at different conductance levels. Hydrogen Bonds (HB) and Salt Bridges (SB) were calculated using the Python package GetContacts (n.d.) with default parameters. The Solvent-Accessible Surface Area (SASA) of each residue in the interacting interfaces formed between two subunits of GluA2 AMPAR and one subunit of the STG (GluA2:STG interface) was also calculated following a protocol similar to that reported by Magalhães et al. (2019), splitting by interacting proteins: ligand (STG) and receptor (two of the GluA2 subunits, Figure 1). These analyses were performed considering the entire complex, without STG and GluA2, for every replica of the three systems to calculate the differences in SASA values (∆SASA) for each residue following Equation 1, where SASA of each residue from GluA2:STG (SASAComplex) was subtracted from SASA of each residue from GluA2 or STG (SASAGluA2/STG).


(1) $$\Delta SASA=\Delta SASA_{GluA2/STG}-\Delta SASA_{Complex}$$

In addition, we calculated the difference in ∆SASA between the WT and mutant systems to assess the effect of the mutation following Equation 2 where SASA of each residue from GluA2:STG (SASA_Complex_) was subtracted from SASA of each residue from GluA2 or STG (SASA_GluA2/STG_). In addition, we calculated the difference in ∆SASA between the WT and mutant systems to assess the effect of the mutation.


(2) $$\Delta \Delta SASA=\Delta SASA_{V143L}-\Delta SASA_{WT}$$

Free binding energy calculations were performed using the AMBER Molecular Mechanics Poisson Boltzmann Surface Area method (MMPBSA; Miller et al., 2012), as implemented in the gmx_MMPBSA package (Valdés-Tresanco et al., 2021). Binding energies (ΔG_Binding_) were estimated using the following formula:


(3) $$\Delta G_{Binding}=G_{GluA2:STG}-G_{GluA2}-G_{Stargazin}$$

Each energy term (Gx) in Equation 3 can be estimated using Equation 4:


(4) $$G_{X}=E_{MM}+G_{solvation}-TS$$

where $E_{MM}$ is the Molecular Mechanics (MM) potential energy in vacuum, which can be obtained as the sum of the bonded and non-bonded interactions (Equation 5):


(5) $$E_{MM}=E_{bonded}+\left(E_{vdW}+E_{elec}\right)$$

$E_{bonded}$ corresponds to the bonded interactions consisting of bond, angle, dihedral, and improper interactions. The van der Waals ( $E_{vdW}$ ) and electrostatic ( $E_{elec}$ ) interactions, which constitute non-bonded interactions ( $E_{non-bonded}$ ), were modelled using the Lennard-Jones (LJ) and Coulomb potential functions, respectively.

$G_{solvation}$ is the free energy of solvation and is defined as the energy required to transfer a solute from vacuum into the solvent. The MMPBSA method uses an implicit solvent model to calculate the energy term (Equation 6):


(6) $$G_{solvation}=G_{polar}+G_{non-polar}$$

Temperature (T) and entropy (S) denote the entropic contribution to the free energy in vacuum, which, owing to the nature of the calculation and thriving on error cancellation, is negligible (Wang et al., 2022). The parameters for the Poisson-Boltzmann calculation were used as default except for the ionic strength, which was set as 0.15 M, the membrane dielectric constant, which was defined as 7, and the membrane thickness, which was set to 39 Å. The radii from the simulation topology files were used in PB and nonpolar calculations. To compare the energy differences between the WT and mutant systems, ΔΔG was calculated using Equation 7.


(7) $$\Delta \Delta G=\Delta G_{V143L}-\Delta G_{WT}$$

All data processing and visualization were performed using in-house R scripts (R Core Team, n.d.; R version 4.1.2).

## 3 Results

The recent study by Yelshanskaya et al. (2022) showed that AMPAR bound to STG are gated to submaximal conductance levels and suggested that the size of the AMPAR gate opening, and the hydrophobicity of the pore constriction are signatures of different conductance states of the receptor. The authors showed that certain structures, such as NNNN and GNNN, may represent a nonconducting state, whereas other structures, such as GNGN1, GNGN2, GGNN, GGGN, and GGGG, may represent the first conductance level (O1) and the 5WEO structure represents the second conductance level (O2). However, several structures such as GNGN1, GNGN2, and GGGG can achieve a conductance level (O2) for a significant fraction of time (Yelshanskaya et al., 2022).

### 3.1 Interfacial contact area and solvent exposition

When calculating the average interface area between STG and AMPAR GluA2, we observed that the nonconducting (NC) structures and GGGG had a higher average interface area in the WT than in the mutated system. An example of this was the NNNN structure, which had an average interface area of 2327.7 Å^2^ per STG without mutation (WT) and 2278.4 Å^2^ per STG with the mutation (V143L). Conversely, the O1 and O2 structures have lower average interfacial areas per interface in the WT system. Of those, the structure GGGN had the biggest difference with an average interface area of 1992.57 Å^2^ per STG molecule in the WT complex and 2354.41 Å^2^ per STG molecule in the mutated systems.

**Figure 2:**
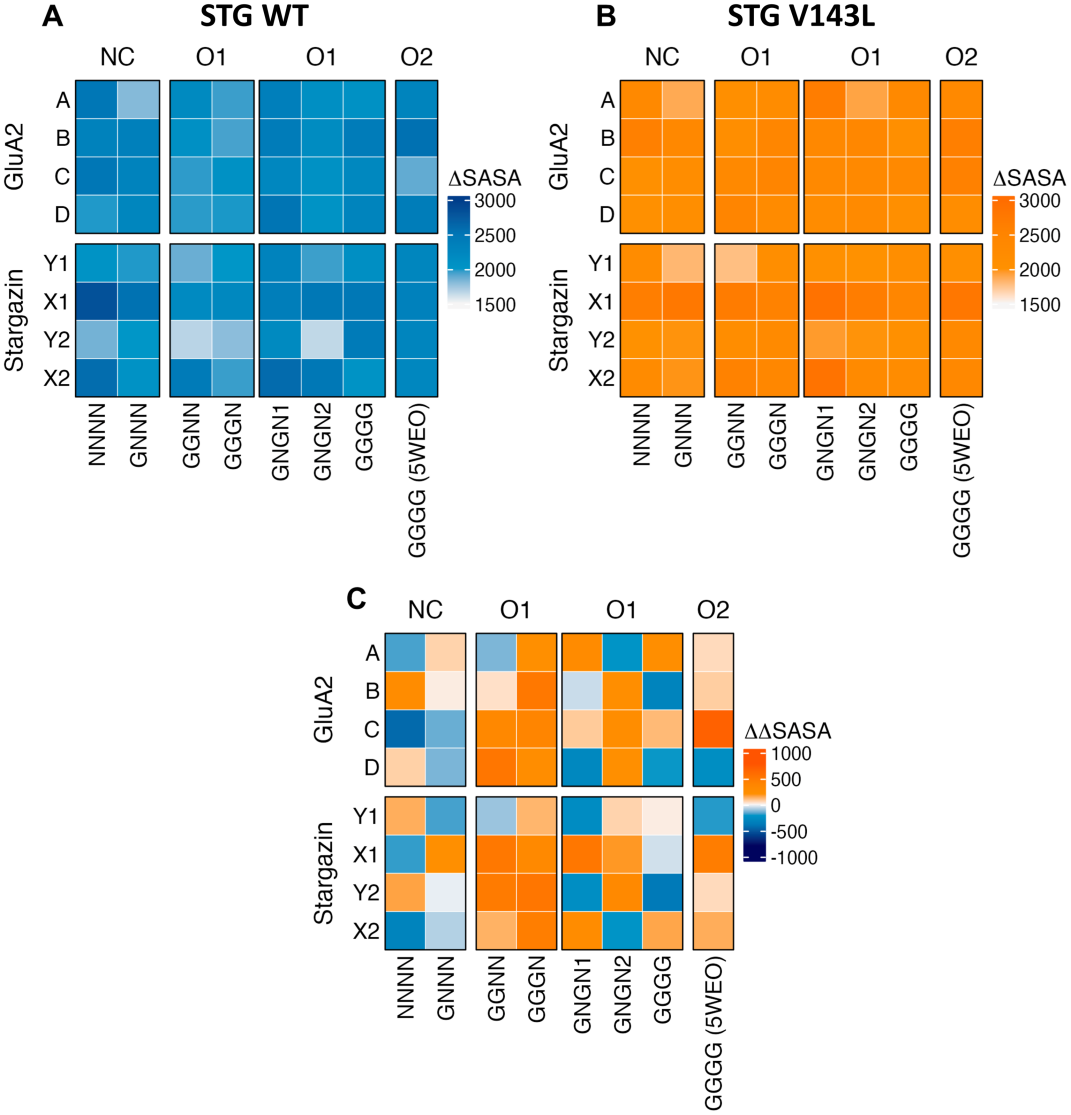
Heatmaps of ΔSASA per protein in the WT **(A)** and V143L **(B)** AMPAR:STG systems, and ΔΔSASA (ΔSASA_V143L_ − ΔSASA_WT_) per protein **(C)**. The values were expressed in Å^2^.

To identify the most relevant residues for the interface between STG and AMPAR GluA2, ∆SASA for each residue was calculated for each frame in each simulation across all simulations (Figures 2A,B). A positive ∆ SASA value indicates that the residues were more occluded from the solvent in the complex than in the isolated components. This suggests that these residues are more buried or shielded within the complex structure, potentially due to protein-protein interactions or conformational changes induced by complex formation. AMPAR monomers with the highest occlusion upon complex formation from NC WT systems were monomers A and C in the NNNN structure, and B and C in the GNNN structure. In the O1 and O2 structures, with the exception of GGGN, the monomers with the highest occlusions were monomers B and D, respectively. This observation highlights the shift that conductance can have in the interface dynamics between AMPAR subunits and STG. When mutated, this shift disappeared, making monomer B the region with the highest shielding across all structures except for GGNN and GGGG. Moreover, our findings indicate that STG binding to the Y site consistently results in higher occlusion than STG binding to the X site, as observed across all structures and systems. This intriguing asymmetry sheds light on the site-specific preferences of the STG in modulating interface dynamics.

At the substructure level (Supplementary Figures SI 1 and SI 2), M1 and M4 showed the greatest protection against complex formation for all AMPAR residues in all structures of both STG WT and V143L systems. B and D (the Main GluA2 of Y site) formed more contacts in LBD_D2 than in A and C. The scale of occlusion for M1 and M4 did not show significant changes between the various GluA2 levels within the structure or when comparing the structures with different conductance levels.

For the STG, the substructures with the largest blocked regions were TMD3 and TMD4 in all systems. The ΔSASA values for TMD3 and TMD4 did not show significant changes between the different STG molecules within the structure or when comparing structures with different conductance levels. The TMD3-β5 loop had a larger occlusion at the Y site.

When comparing ΔΔSASA (ΔSASA_V143L_ − ΔSASA_WT_) per protein (Figure 2C), we assessed how the V143L mutation affects the accessibility and exposure of residues in the protein structure and their interface area, which can provide valuable insights into its structural and functional consequences. A positive ΔΔSASA suggests a potentially larger interface area upon V143L mutation, whereas a negative ΔΔSASA suggests a smaller interface area in the mutant. We conclude that the WT system nonconductive structures have a larger interfacial area than V143L, particularly the Y-site STG and monomer A in the NNNN structure. The structures in the O1 and O2 states have a wider interface in V143L, except for the GGGG structure.

At the substructure level (Supplementary Figure SI 3), we observed that M1 and M4 from NNNN and GNNN tended to exhibit a broader interface area in the WT system; thus, a greater effect was observed for the residues of this system. Structures in the O1 and O2 states tend to have smaller interface areas in the WT system, with some regions having similar values. LBD_D2 showed the reverse pattern: the WT nonconductive structures had smaller interfacial regions, whereas the WT O2 structure and GGGG were involved in the formation of larger interfaces compared to the mutant system. When comparing the ΔΔSASA values of STG, the pattern was less pronounced (Supplementary Figure SI 3). However, for one of the X-sites, we observed that TMD3_β5 had a significantly larger contribution to the interface area in the WT system, whereas this effect was negligible at the Y sites and at another X-site for the NC and O1 states. At the Y-site, the TMD3 region exhibited a reduced contribution to the interface in the WT complex, highlighting a distinct spatial interaction pattern compared with other sites.

### 3.2 Key interfacial pairwise interactions

#### 3.2.1 Hydrogen bonds

**Figure 3:**
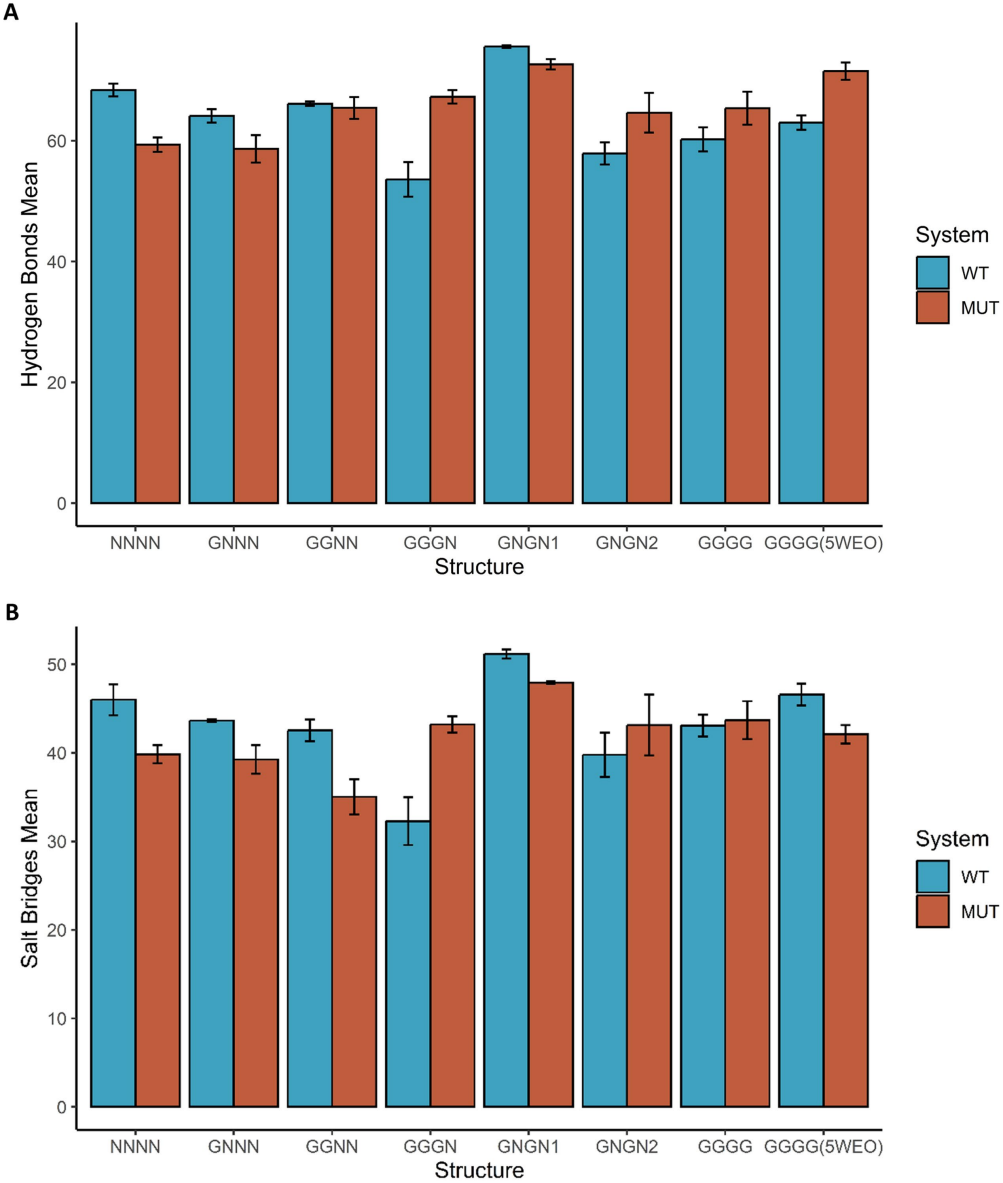
Hydrogen Bonds and Salt Bridges in GluA2:STG Complex Structures. The average number of hydrogen bonds **(A)** and salt bridges **(B)** formed between GluA2 and STG in the eight states are coloured blue in the WT systems and dark orange in the systems with the mutation STG V143L. Error bars were calculated using the standard deviation of the number of bonds formed during the simulation.

Figure 3 shows the average number of Hydrogen Bonds (HB) and Salt Bridges (SB) formed between STG and the two AMPAR subunits. Notably, we observed that nonconductive structures have a decrease in the number of HB formed in the presence of the V143L mutation; for example, in the NNNN-WT structure, the average number of HB formed was 68.40 ± 1.05, while in the NNNN-V143L system 59.35 ± 1.19 HB were formed. In the O1 and O2 states, apart from GNGN1 and GGNN, the mutation increased the number of HB formed. For example, in the 5WEO structure, the average number of HB formed in the WT system was 63.01 ± 1.19, while in the V143L system 71.53 ± 1.44 HB were formed.

The occupancy of these interactions throughout the MD simulations was also calculated and is presented as a heat map (Supplementary Figures S4–S11). In all structures, we observed that the Y site tended to form more HB than the X site. Additionally, we can again observe that the NC structures tend to form fewer HB structures than the other structures and that the WT NC structures tend to form HB with higher occupancy than their mutated counterparts. In O1 structures, the occupancy of HB tended to increase when the structures were mutated, except for GGNN. In the structure in the O2 state, we observed that the V143L mutation slightly increased the HB occupancy. Although the residues involved in the formation of HB tend to be similar in every structure, few HB are present in all structures with a high frequency. A good example of an HB formed at both the X and Y sites in all the structures is between Glu95 (ECH) and Lys532 (LBD-M1 loop). The NC structures showed a decrease in occupancy, whereas most O1 structures (except GGNN) showed a tendency to increase occupancy of the X site. Additionally, the GGGG structure showed a considerable increase in the number of Y sites. The O2 structure was barely affected by the mutation with respect to occupancy of this interaction. Another important HB was formed between Tyr176 (β5) and Glu545 (M1). The NC structures showed a decrease in occupancy, especially at the X site, whereas the O1 structures, with the exception of GGNN, showed an increase in occupancy. Remarkably, the V143L mutation did not allow the GGNN structure to form HB. Regarding the structure in the O2 state, we observed a decrease in occupancy, particularly at the Y-site.

#### 3.2.2 Salt bridges

Regarding the effect of the mutation on the formation of SB (Figure 3B), in NC and O2 structures, the mutation decreased the number of SB; for example, the NNNN structure formed an average of 45.99 ± 1.76 SB in the WT system and 39.83 ± 1.02 SB in the V143L system. In O1 structures, the pattern was less evident, but in the majority the mutation allowed the formation of more SB compared to the WT, for example in the GGGN system, in which the average of SB formed was 32.28 ± 2.69 in the WT system and 43.21 ± 0.93 in the V143L system. However, in GNGN1 and GGNN structures, the mutation decreased the formation of SB; for example, GGNN formed 42.51 ± 1.22 SB in the WT system and 35.03 ± 2.00 in the V143L system.

The occupancy of these interactions throughout the simulation was quantified and is depicted as a heatmap (Supplementary Figures S12–S19). In line with HB observations, the Y site typically showed a higher propensity for SB formation than the X site. Furthermore, NC structures generally exhibited lower SB occupancy when they contained the V143L mutation. In contrast, for structures in the O1 state, SB occupancy tended to increase with mutations, except for the GGNN and GGGG configurations. However, in the O2 state, the V143L mutation leads to a decrease in SB occupancy, particularly at the X site.

A consistently high-frequency SB was observed between Glu95 (ECH) and Lys532 (LBD-M1 loop) across the various structures. In NC structures with the V143L mutation, SB occupancy decreased, particularly at the X site. In O1 state structures, excluding GGNN and GGGG, the presence of the mutation was associated with increased SB occupancy. Specifically, the GGNN structure ceased to form an SB at the X site with the mutation, whereas the GGGG structure showed a marked decrease in the SB frequency, particularly at the Y site. Conversely, in the O2 state, there was a notable increase in the SB occupancy, particularly at the Y site. This detailed analysis helps to elucidate the differential impact of the V143L mutation on salt bridge dynamics across various states and sites of the receptor.

### 3.3 Energetic analysis of the main protein-protein interactions

**Figure 4:**
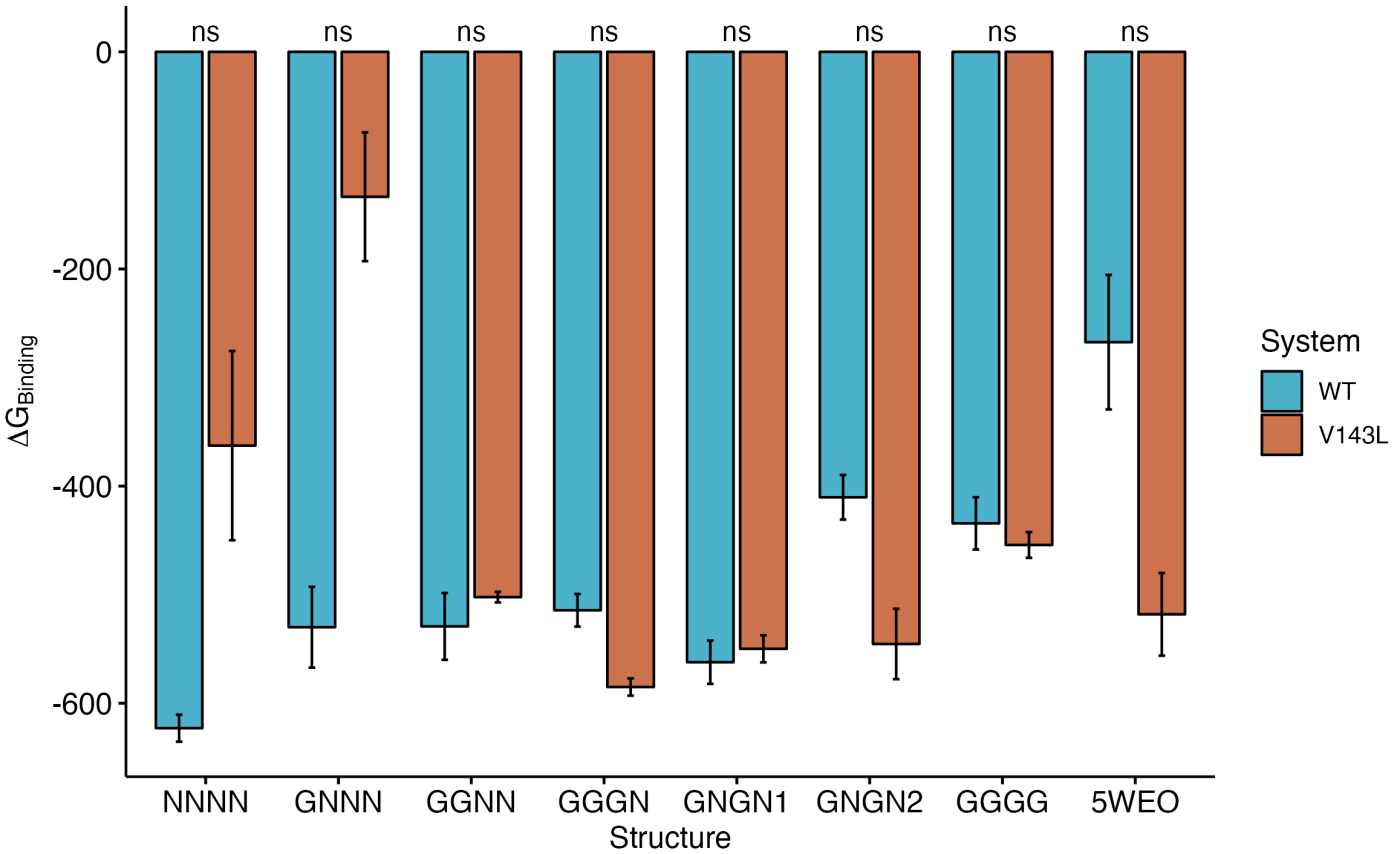
∆G_Binding_ for STG WT and STG V143L GluA2:STG complexes. The values are expressed in kcal/mol. Error bars were calculated using the standard deviation of ∆G_Binding_ during the simulation. The *p* values between the WT and STG V143L systems were calculated using the Wilcoxon test.

The total ∆G_Binding_ values of the various Protein-Protein Interactions (PPIs) exhibited different patterns, depending on the conductance level (Figure 4). NC structures were significantly more stable in the WT system than in the V143L system (lower ∆G_Binding_ values in the WT system). Systems in the O1 state displayed similar stabilities in both the WT and V143L mutated forms, with the exception of GNGN2 and GGGN configurations. These specific configurations showed increased stability when mutated, which aligns with previous observations that they can sometimes achieve a conductance level that is characteristic of the O2 state. As such, in the O2 state, the interactions were significantly stronger in the mutated system than in the WT, further strengthening the role of the V143L mutation in enhancing stability and binding interactions in this particular state.

The ∆∆G values were determined (Supplementary Figure SI 20) to elucidate the effects of the mutations on the stability of the protein complex. A positive ∆∆G value indicates that the mutation renders the system less stable than that of the WT, typically because of a reduction in binding affinity. In contrast, a negative ∆∆G value suggests that the mutation enhanced the stability of the system relative to that of the WT, often as a result of improved binding affinity. The STG subunits within the complexes did not exhibit large absolute ∆∆G values, indicating minor mutation-induced differences in the stability. However, the STG proteins in the NC structures demonstrated greater stability in the WT system than those with the V143L mutation, as reflected by the higher ∆∆G values. Conversely, in some O1 and O2 state structures, the interactions were stronger in the V143L system, suggesting enhanced stability due to the mutation. The primary distinction between the WT and mutated systems was driven by the contribution of GluA2, notably in chains B, C, and D. Here, we noted a clear variability in stability, highlighting the varying impact of the mutation across different subunits and functional states. The most pronounced differences were observed at the extremes of the conductance. In the NC structures, GluA2 interactions were markedly more stable in the WT systems, particularly in chain B in NNNN, and chains C and D in GNNN. In the O2 state structures, chains A and B demonstrated stronger interactions in the WT, whereas chains C and D exhibited stronger interactions in the V143L system, indicating a varied response to the mutation, depending on the specific chain and state of conductance.

The contribution per substructure was analysed to further understand the ∆G differences (Supplementary Figure SI 21). Specifically, focusing on GluA2, the V143L mutation decreased the interaction strength of LBD-D2 in both NC and O2 state structures. Regarding the STG substructures, the ECH and loop between TMD3 and β5 showed decreased stability in the interaction between the NC and O1 states due to the mutation. However, in the O2 state, an increase in the interaction strength was observed. In TMD3 of STG, the V143L mutation reduced the interaction strength in NC structures, but enhanced it in both O1 and O2 structures, with the exception of GNGN1 structures.

We further calculated ΔG for all residues, distinguishing between STG and GluA2, as well as between mutant and WT complexes (Supplementary Figures SI 22–25). Additionally, we analysed the average ΔΔG values for all residues within GluA2 and STG across various states (Supplementary Figures SI 26, 27). Focusing on specific residues of STG, Supplementary Figures SI 23 and SI 25 reveal that, while most residues did not display significant changes in ΔG, Glu95 and Ser158 were less stable in the O1 and O2 structures. In the mutated systems, Ser165, Lys166, and Ser167 were more stable in O1 and O2 structures than in NC structures. The main differences in ΔG between the wild-type and mutated STG, as shown in Supplementary Figure SI 27, indicated that Leu3, Phe4, Glu95, Pro164, Ser165, and Lys166 were generally more stable in the mutated systems in O2 structures, whereas in NC structures, they were more stable in WT systems. Notably, Glu90 in the GNGN1 system showed significantly more stable interactions in the WT, particularly in chains B and C.

For GluA2 residues, as detailed in Supplementary Figures SI 22 and SI 24, Glu591, Glu655, Ser717, and Tyr818 exhibited lower stability in O2 than in O1, whereas Asp798 showed increased stability. In the mutated systems, Glu587 and Glu648 showed decreased stability in most conductive structures, particularly GGGN, GGGG, and 5WEO. The differences in ΔG for GluA2 between the wild-type and V143L mutated systems, outlined in Supplementary Figure SI 26, showed that the NC structures displayed stronger interactions with Lys530 and Lys532 in the WT system than in the O2 structure. In chain A of the NNNN system, residues from the LBD_M4 loop (Ser799 to Lys804) exhibited lower stability in the mutated system than in other configurations such as GGGG and 5WEO.

### 3.4 Pore radius

**Figure 5:**
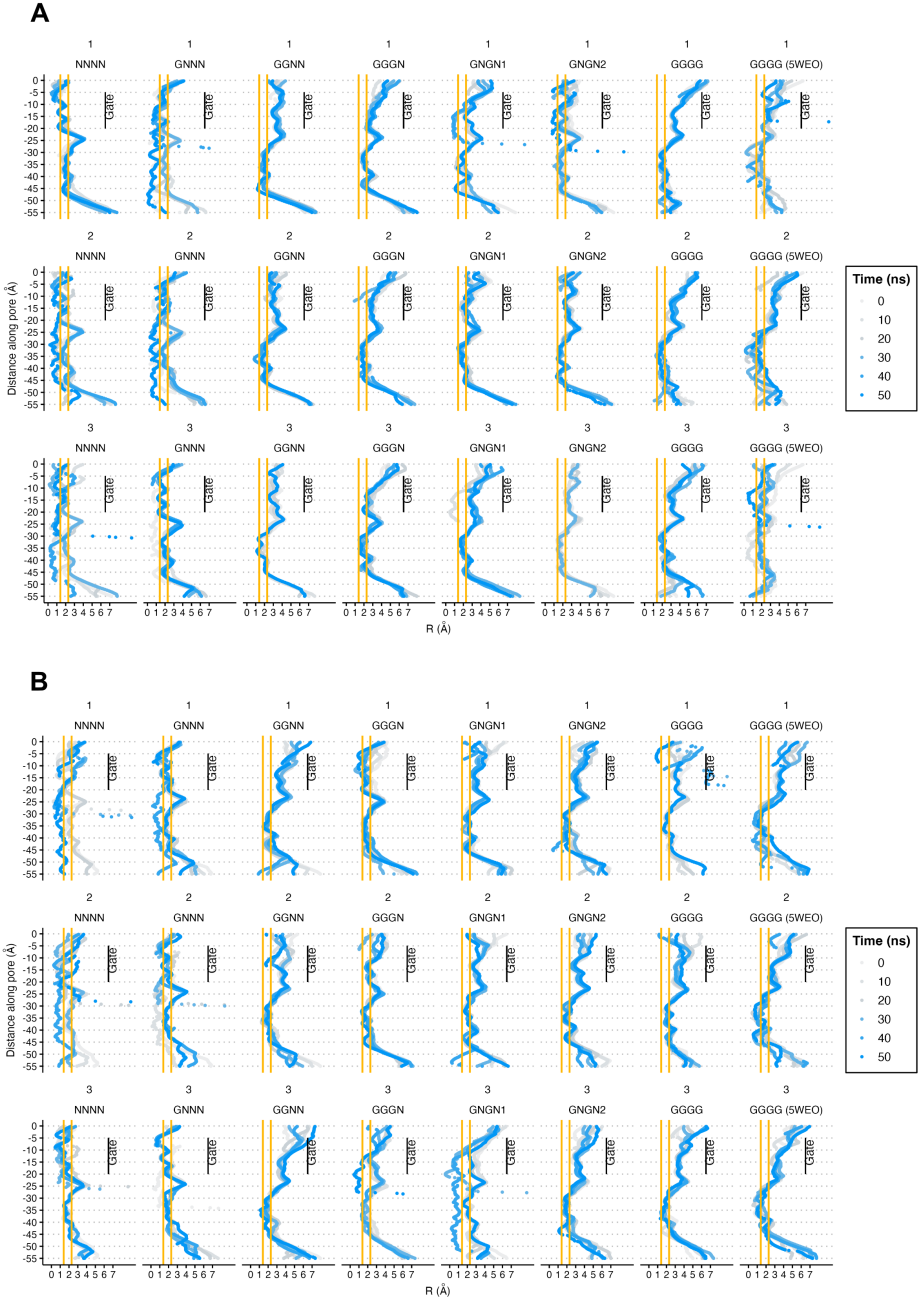
Pore radii of STG WT **(A)** and V143L **(B)** GluA2:STG complexes. The orange lines at 1.4 and 2.3 Å help identify between non-conductive and O1 and O2 conductance level for each AMPAR (Yelshanskaya et al., 2022). The values were calculated at intervals of 10 ns and expressed in Å.

Following the description by Yelshanskaya et al. (2022), we performed pore radius analysis for both the WT and V143L mutant systems (Figure 5). Our observations revealed that, in both the WT and V143L systems, the NC structures did not transition to an open conformation during the simulations of either system. This suggests that a single glutamate molecule interacting with AMPAR is insufficient for opening the gate. In contrast, for structures in the O1 and O2 states, the V143L mutation appeared to facilitate higher conductance levels, as evidenced by the generally larger pore radii in these mutated systems.

## 4 Discussion

The recent release of cryo-EM structures of AMPAR:STG complexes has enriched our understanding of intricate interactions between these two proteins, revealing their dependence on structural motifs and conductance states. Specifically, the binding site of STG spans two GluA2 subunits within the tetrameric GluA2 channel, where the M1, M2, and M4 domains of one GluA2 subunit interface with the TMD3 and TMD4 of STG. Through MD simulations, we gained deeper insights into the stability of this interface and provided a detailed atomic-level characterisation of the interactions between STG and GluA2, both with and without the clinically significant STG V143L mutation.

Our study highlights several key factors essential for PPI formation, including hydrogen bonding and salt-bridge dynamics. Energetic analyses of interfacial residues revealed their roles in maintaining a functional complex, while ∆SASA values shed light on the dynamic residue exposure at the interface. An examination of the key interfacial contacts revealed that the average number of HB and SB formed during the simulation of nonconductive systems was lower in the mutated systems, whereas the opposite trend was observed in conductive systems. This pattern suggests a potentially adverse effect of the V143L mutation on the stability of the interactions between STG and AMPAR in the NC state. The ∆SASA values also suggest that the STG V143L mutation tends to diminish the interface area in nonconductive structures while expanding it in conductive states. Additionally, STG bound to the Y site consistently showed higher ∆SASA values than the X site in all structures and systems, which is consistent with our previous results (Caldeira et al., 2022). In that study, we found significantly decreased GluA surface and synaptic levels in the presence of the STG V143L mutation (Caldeira et al., 2022). This mutation also reduced the STG synaptic residence time and significantly affected synapse morphology, synaptic connectivity, and plasticity in the basal dendrites of CA1 hippocampal neurons.

Remarkably, this dual effect in the nonconductive vs. conductive AMPAR:STG states was further reflected in the ∆Gbinding values: lower for WT in the NC state but reduced for the V143L mutant in the conductive state, implying that the mutation disrupts interactions in the inactive state while stabilising the complex in the active state. The finding that the STG V143L mutation disrupts STG-AMPAR interactions in the inactive GluA2 state implies that the mutation could interfere with GluA2 transport to synaptic sites, as observed in our previous study (Caldeira et al., 2022). This result in the NC structures indicated that the influence of the mutation may extend to the dissociation of STG from AMPARs before channel activation. Indeed, we previously found that the co-immunoprecipitation of GluA1 with STG was reduced in the presence of STG V143L (Caldeira et al., 2022). Moreover, the mutation may interfere with STG dissociation from the AMPAR complex, as STG interaction with AMPAR has been associated with reduced decoupling linked to desensitisation (Shaikh et al., 2016).

Latsko et al. (2022) described a *de novo* missense variant of GluA2 (L530M) associated with global developmental delay, autism spectrum disorder, and epileptic encephalopathy. This specific residue consistently demonstrated low ∆G_binding_ values across all studied structures. Notably, this variant was positioned near a region of high interaction frequency between Glu95 in STG ECH and Lys532 in GluA2. These residues are crucial for the stability of the STG-GluA2 interface, and substitution of a positively charged hydrophobic amino acid near Lys532 likely alters the position of this residue, which may disrupt its interaction with Glu95. Hawken et al. (2017) explored the functional effects of several GluA2 mutations and their impact on binding with STG and cornichon 3, highlighting important contributors to the functionality of TARPs and their interaction with GluA2. Notable mutations, such as C549L, L810F, and A814F, maintain normal STG binding, but alter the gating properties of GluA2. All these sites showed in our study to have low ∆G_binding_ values and minimal differences between WT and mutated systems, confirming earlier findings (Caldeira et al., 2022). Although these mutations do not change the nonpolar characteristics of the affected region, they introduce bulkier amino acids that might reduce the tightness of the interaction between GluA2 and STG, potentially affecting critical gating functions involving the KGK motif and β1-β2 loop of STG.

Echoing findings of the Yelshanskaya et al. (2022), our simulations demonstrated that both the STG WT and V143L mutant structures successfully reached an O2 conductive state. This finding supports the notion that channel activation requires the interaction of at least two glutamate molecules with LBDs. However, not all four LBDs must be engaged with glutamate for O2 conductance. Interestingly, in the V143L mutant structures, particularly in structures such as 5WEO and GNGN2, the channel gate seems to remain open longer and displays a larger pore radius than the WT, further supporting the hypothesis of altered gating dynamics in the mutant. In a previous study, we found that STG V143L did not cause changes on the amplitude or kinetics of AMPAR-mediated miniature excitatory postsynaptic currents recorded from CA1 hippocampal neurons, but found defective evoked responses specifically in the basal dendrites of CA1 pyramidal neurons, where STG is enriched (Caldeira et al., 2022).

Furthermore, our results indicate that the STG mutation has a dual effect on the AMPAR complex: it reduces stability in the absence of glutamate, but enhances stability when glutamate is bound. This observation suggests an influence of the mutation on AMPAR assembly and trafficking, particularly within the Endoplasmic Reticulum (ER), where glutamate binding may not occur. The ER plays a crucial role in the proper folding and assembly of AMPARs, which is vital for their subsequent transport to the membrane. The destabilising effect of the mutation in the absence of glutamate binding may hinder this process, potentially resulting in increased AMPAR degradation or impaired transport. This can lead to a reduction in the number of functional receptors that reach synapses. Such an effect would directly affect the availability of synaptic AMPARs, potentially causing significant alterations in the synaptic strength and plasticity. Although the mutation may impede receptor maturation and transport, it seems to boost receptor stability and functionality when glutamate is bound, possibly extending the receptor’s open state or diminishing its susceptibility to desensitisation. This increased stability could result in extended Excitatory PostSynaptic Potentials (EPSPs), thereby enhancing synaptic efficiency during periods of receptor activation. Consequently, the mutation may exert a two-fold effect, interfering with receptor trafficking, while simultaneously improving the functional characteristics of AMPARs at the synapse.

The influence of this mutation may extend to the dissociation of STG from AMPARs following channel activation. Prior research has indicated that stargazin detaches from the receptor upon channel opening (Morimoto-Tomita et al., 2009), a process that is potentially affected by STG mutations. Should the mutation hinder this detachment, it could significantly affect the short-term synaptic plasticity.

These observations underscore the intricate and situation-specific effects of this mutation on AMPAR regulation, with potential ramifications in synaptic function and plasticity. While receptor destabilisation in the endoplasmic reticulum may disrupt AMPAR assembly and trafficking within the ER and reduce the surface expression of functional receptors, especially in the synapse, enhanced membrane stability of the STG:AMPAR complex could alter synaptic transmission by fostering a more sustained glutamate response. Moreover, the possibility of modified STG detachment following receptor activation introduces a novel regulatory aspect that may influence synaptic receptor dynamics and mechanisms of plasticity. The biological effect of the STG V143L mutation on the activated STG:AMPAR complex appears to be minimal, especially compared to its effects in non-conductive systems. This suggests that patients with this mutation may benefit from drugs designed to enhance the interaction stability prior to channel activation, similar to the modulators for TARP-γ8 binding to AMPAR, which have recently been described (Zhang et al., 2023). Collectively, these insights shed light on the molecular mechanisms of AMPAR regulation and highlight potential therapeutic targets for conditions involving altered protein-protein interactions.

## Data Availability

The raw data supporting the conclusions of this article will be made available by the authors, without undue reservation.
