# Supplementary material for: Exploring the impact of the stargazin V143L mutation on the dynamics of the AMPA receptor: stargazin complex
Source: Front Cell Neurosci. 2025 Jan 17;18:1505846. doi: 10.3389/fncel.2024.1505846 (PMC11782175; doi:10.3389/fncel.2024.1505846)
Supplement: Supplementary file 1 [file Table_1.DOCX]

Supplementary Material

Exploring the Impact of the Stargazin V143L Mutation on the Dynamics of the AMPA Receptor:Stargazin Complex

Raquel P. Gouveia^1,2†^, Carlos A.V. Barreto^2,3†^, Rita Melo ^4^, Ana Luísa Carvalho^1,2^, Irina S. Moreira^1,2*^

^1^ Department of Life Sciences, University of Coimbra, Calçada Martim de Freitas, 3000-456 Coimbra, Portugal

^2^ CNC - Center for Neuroscience and Cell Biology, CIBB - Center for Innovative Biomedicine and Biotechnology, University of Coimbra, Coimbra, Portugal

^3^ IIIs - Institute for Interdisciplinary Research, University of Coimbra, Coimbra, Portugal

^4^ Centro de Ciências e Tecnologias Nucleares and Departamento de Engenharia e Ciências Nucleares, Instituto Superior Técnico, Universidade de Lisboa, CTN, Estrada Nacional 10 (km 139,7), 2695-066 Bobadela LRS, Portugal

† These authors contributed equally to this work and share first authorship

*** Correspondence:**Irina Moreira
irina.moreira@cnc.uc.pt

***
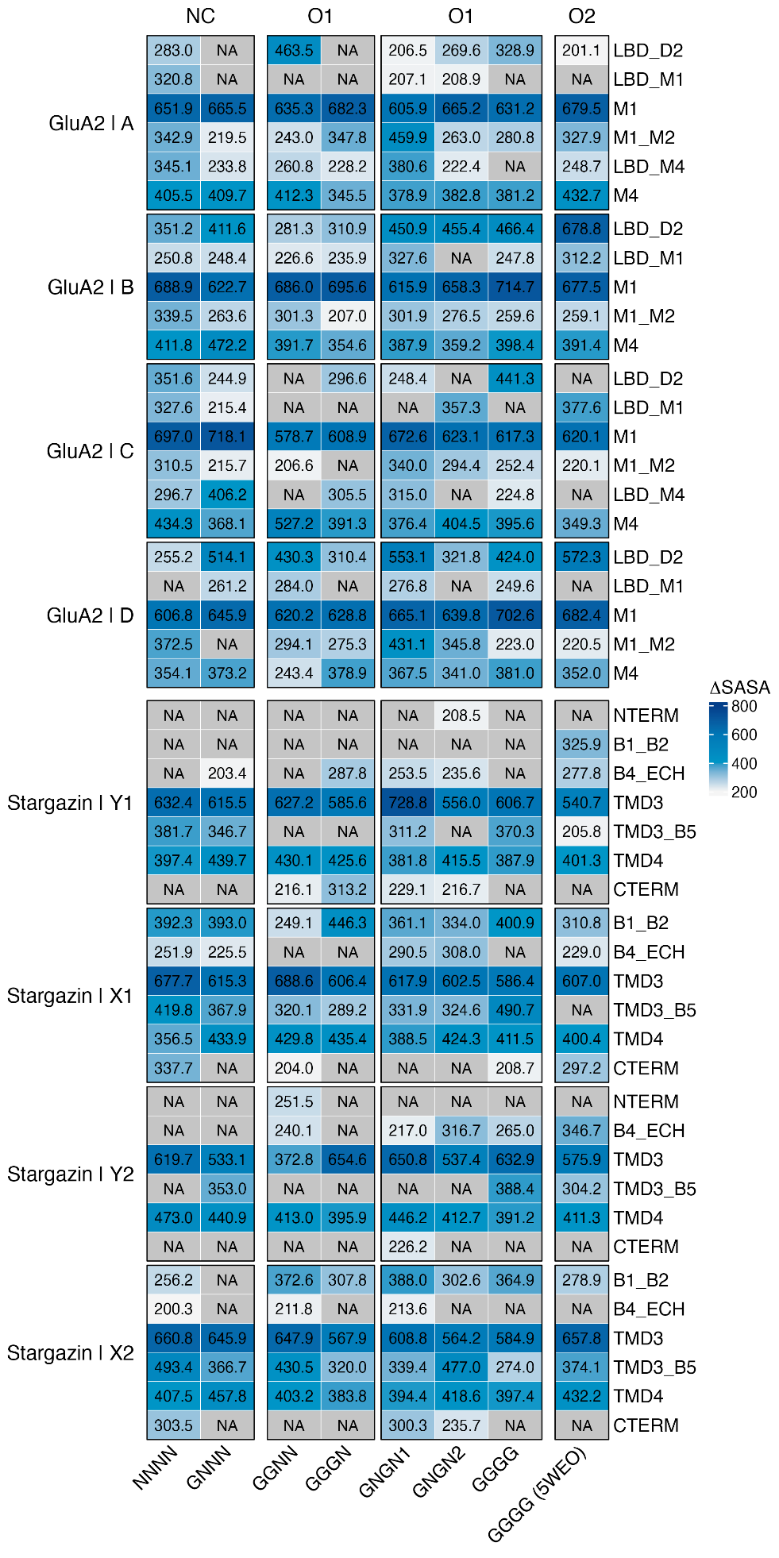
***

**F****ig****ure SI 1. ΔSASA per Substructure for the WT systems.** These values correspond to Å^2^. They were calculated by subtracting the SASA of the monomers (STG and GluA2) from the SASA of the AMPAR:STG complex. The SASA values were obtained using GROMACS. Only substructures with ΔSASA > 200 Å^2^ in at least one of the systems are shown in the heatmap. “NA” means that the ΔSASA value is lower than 200 Å^2^ for that specific system. For GluA2, the M1 substructure had the highest ΔSASA, and for stargazin, the substructure was TMD3. The loop between TMD3 and β5 typically has higher values at the X sites than at the Y sites. Regarding the 2^nd^ domain of the LBD, the O2 state has high values in GluA2 B and D.


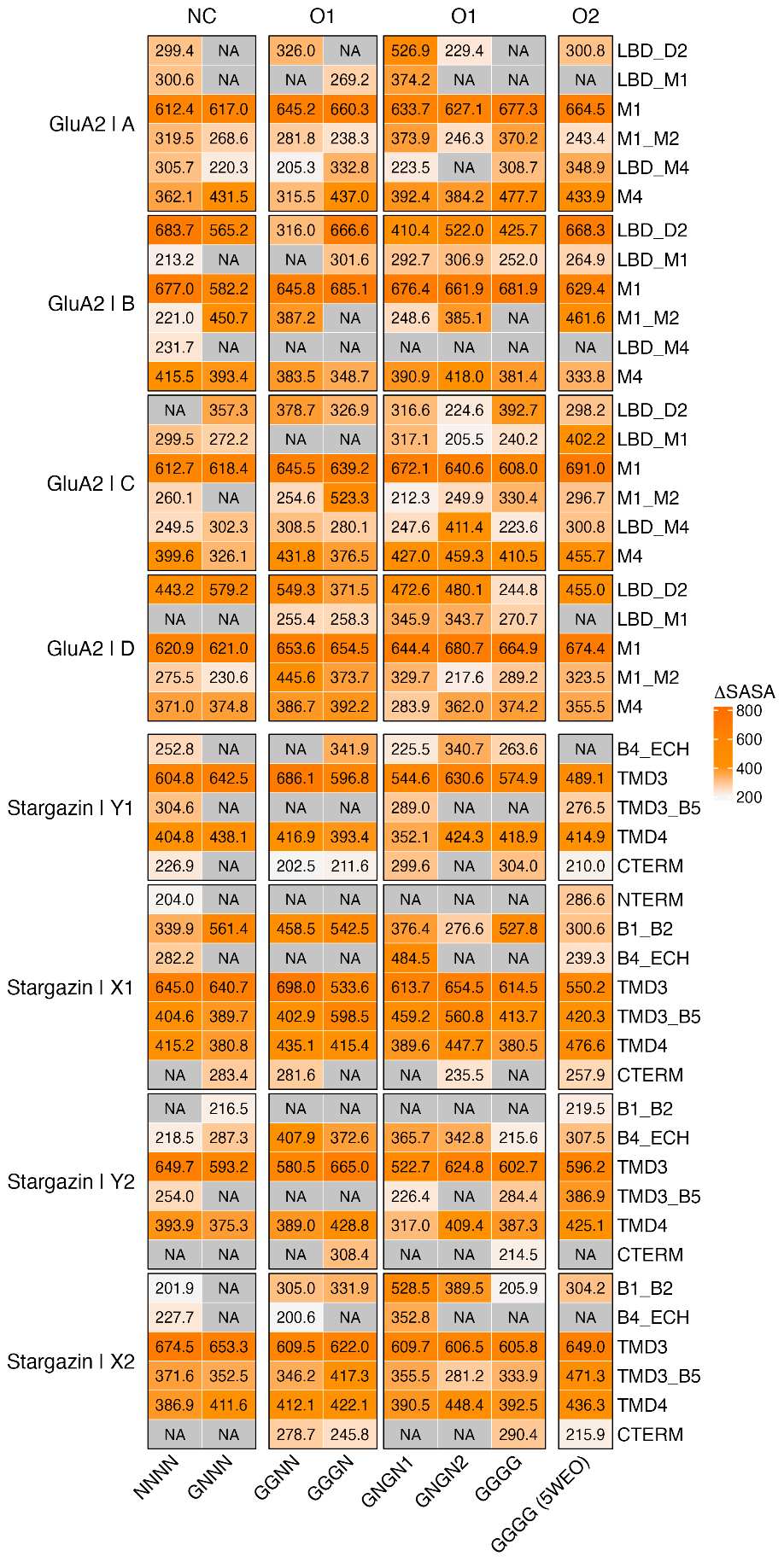


**Figure SI 2. ΔSASA per Substructure for the MUT systems.** These values correspond to Å^2^. They were calculated by subtracting the SASA of the monomers (STG and GluA2) from the SASA of the AMPAR:STG complex. The SASA values were obtained using GROMACS. Only substructures with ΔSASA > 200 Å^2^ in at least one of the systems are shown in the heatmap. “NA” means that the ΔSASA value is lower than 200 Å^2^ for that specific system. For GluA2, the M1 substructure still had the highest ΔSASA, and for stargazin, the substructure was TMD3. The loop between TMD3 and β5 typically has higher values at the X sites than at the Y sites. The 2^nd^ domain of LBD has typically higher values in GluA2 B and D, compared to the other GluA2.

***
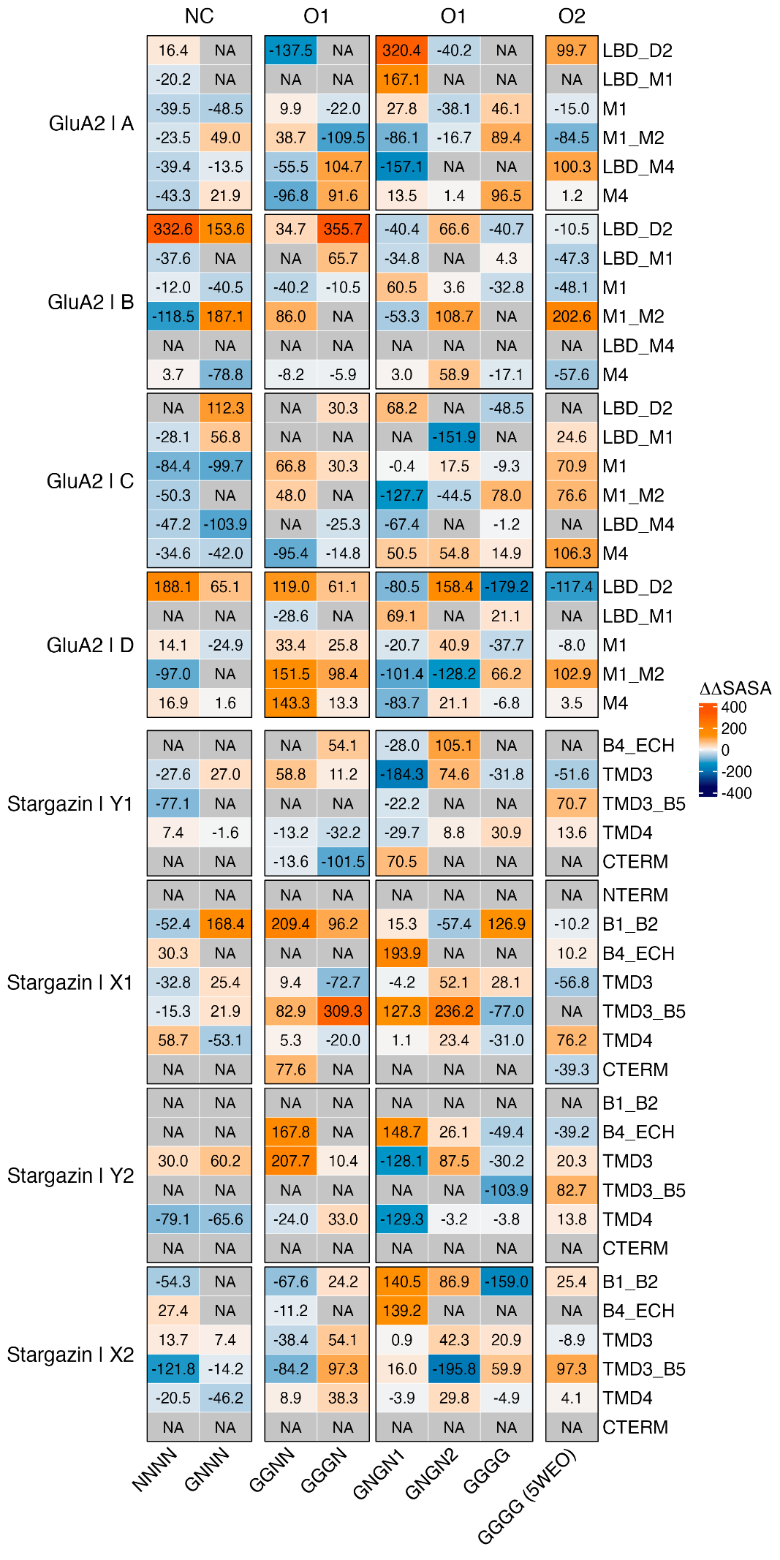
***

**Figure SI 3. ∆ΔSASA per substructure.** These values corresponded to Å^2^ and were calculated by subtracting the values of ΔSASA_V143L_ from the values of ΔSASA_WT_. In the non-conductive states, the 2^nd^ domain of LBD from GluA2 B and D had higher values in the system with STG WT than in the system with STG V143L. Non-conductive states, particularly in the NNNN system, had a larger area in the system with STG V143L.


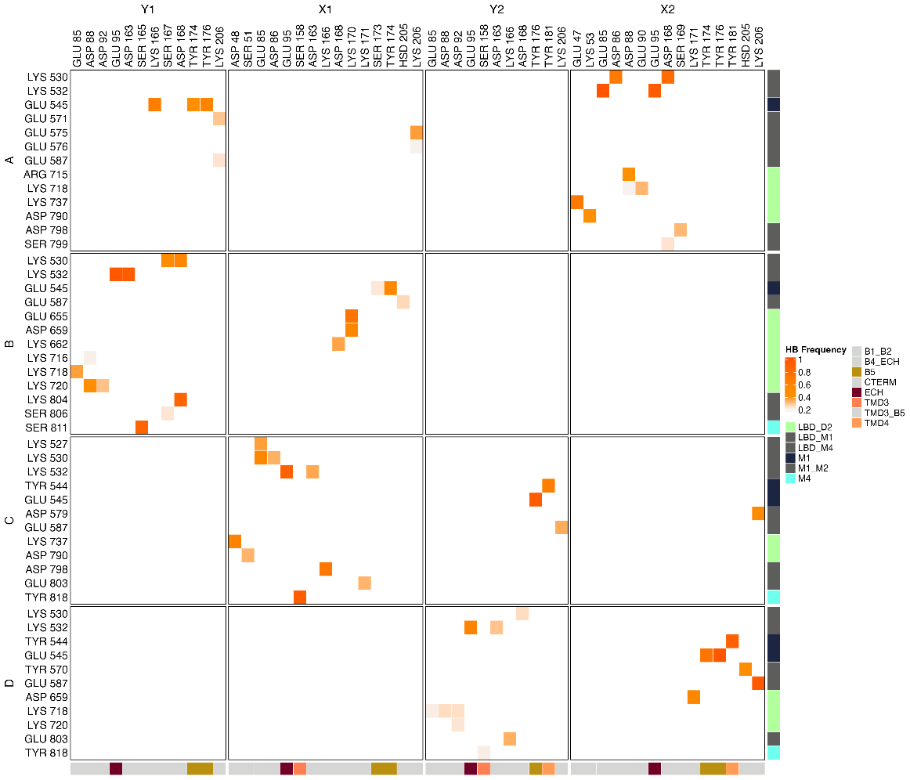

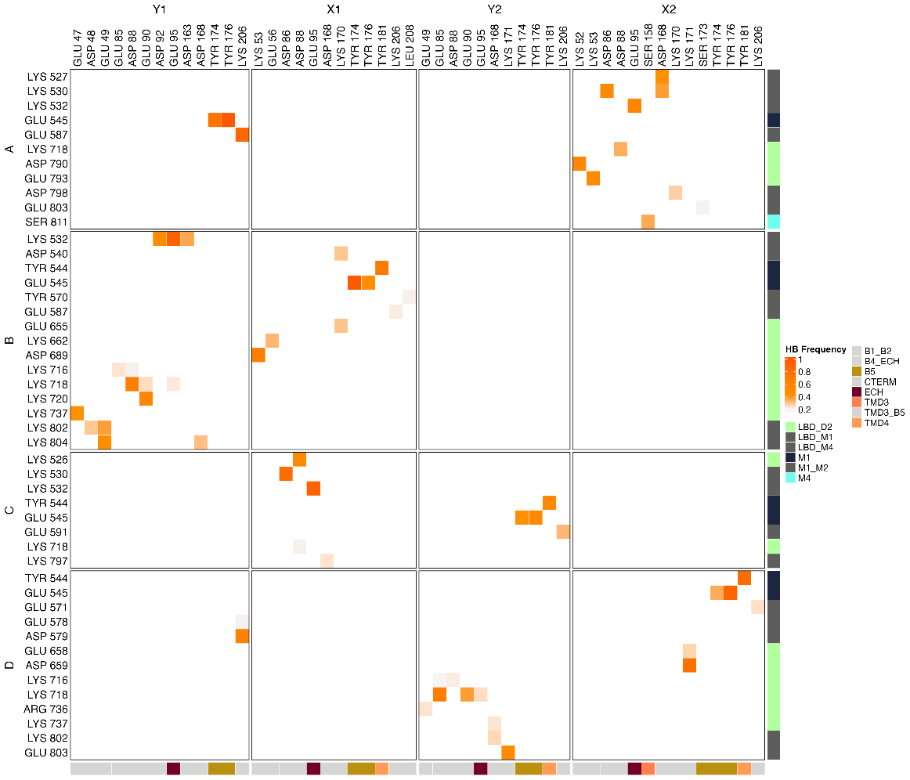


Figure SI 4. Hydrogen bond frequency of complex NNNN per substructure. The HB frequency was calculated using the Python package GetContacts with default parameters. The heatmap at the top corresponds to the NNNN complex with the WT STG, whereas that at the bottom corresponds to the mutated system. Compared with the STG WT system, the number of HBs formed and their occupancy decreased in the presence of the V143L mutation.


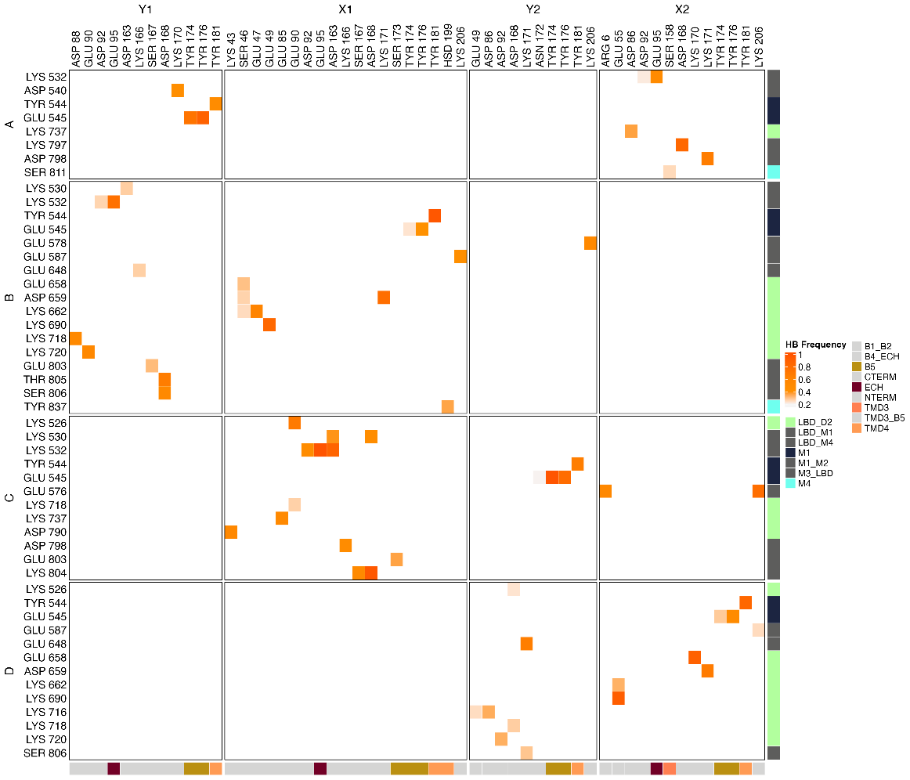

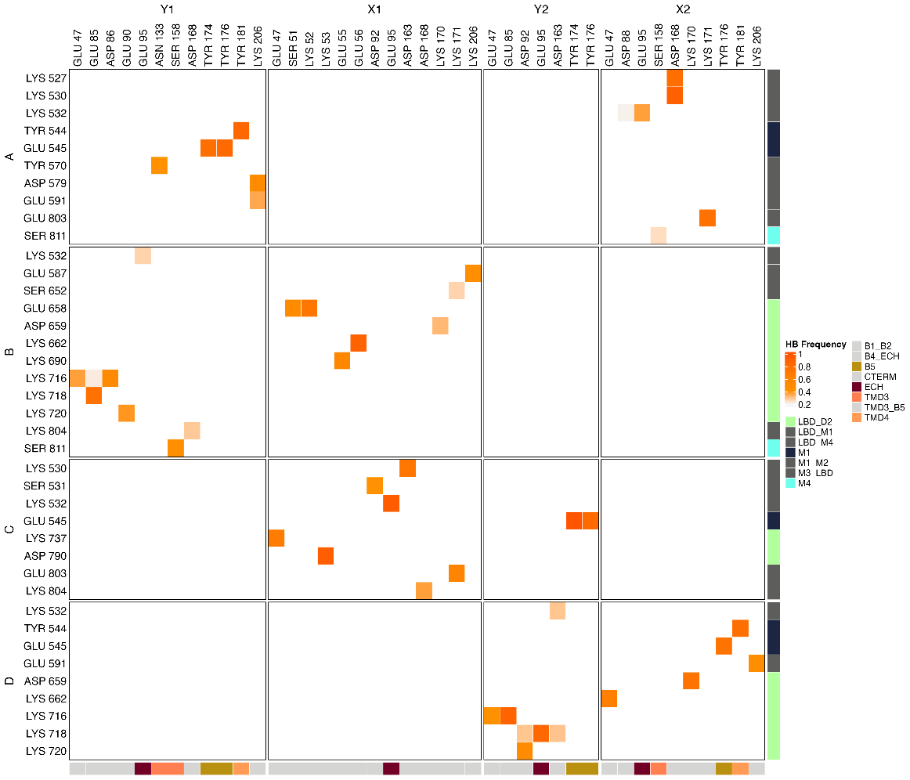


**Figure SI 5. Hydrogen bond frequency of complex GNNN** **per substructure.** The HB frequency was calculated using the Python package GetContacts with default parameters. The heatmap at the top corresponds to the GNNN complex with STG WT, whereas that at the bottom corresponds to the mutated version. Compared with the WT system, the number of HBs formed and their occupancy decreased in the presence of the STG V143L mutation.


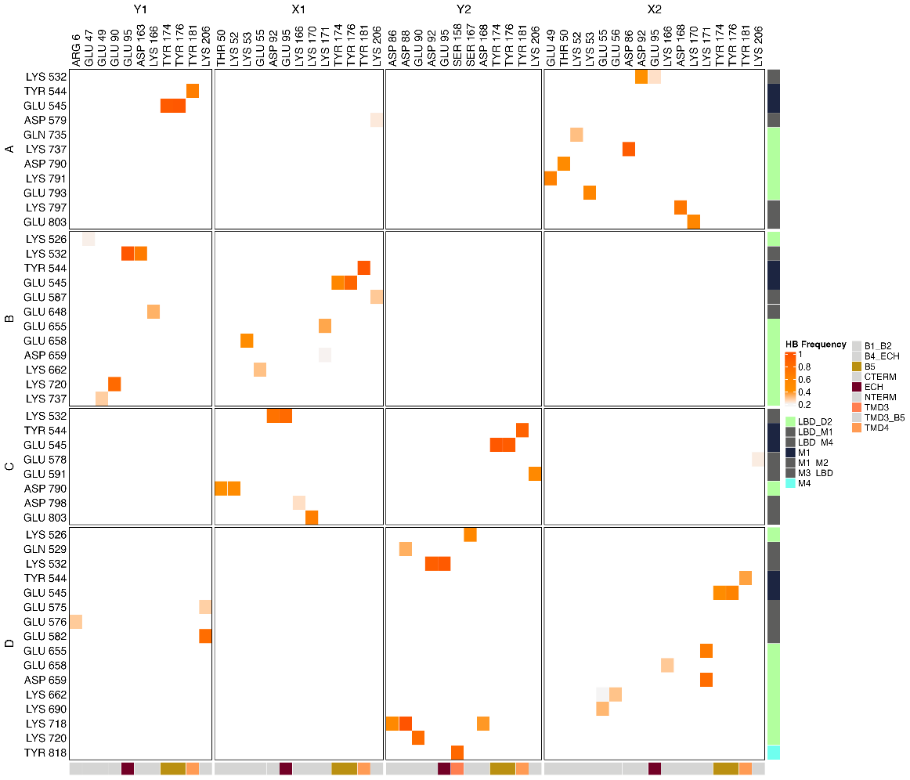

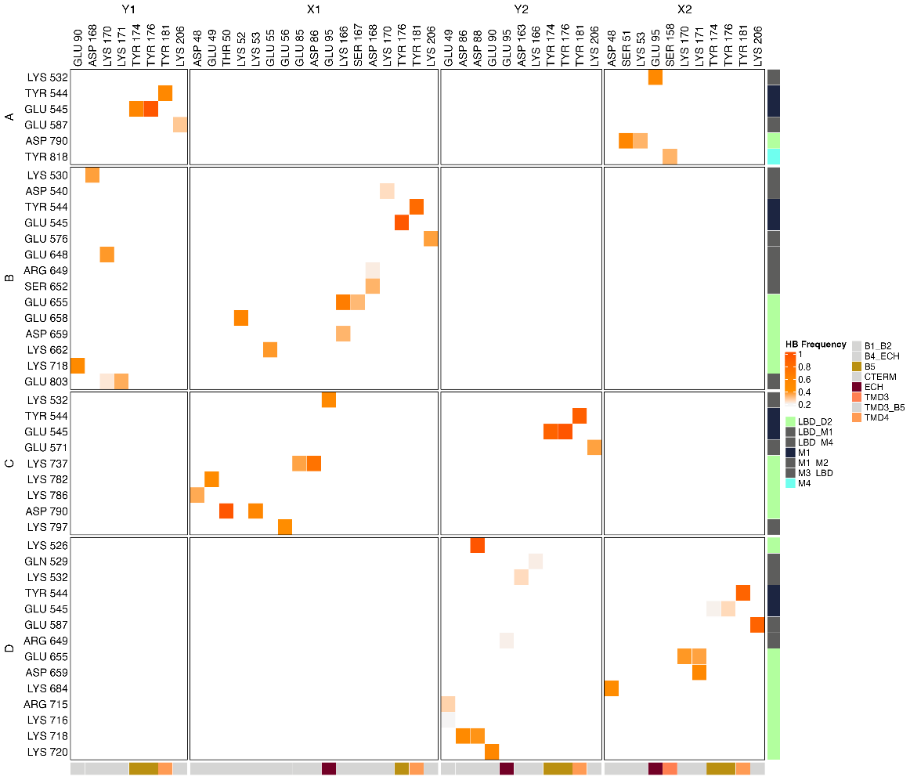


Figure SI 6. Hydrogen bond frequency of complex GGNN per substructure. The HB frequency was calculated using the Python package GetContacts with default parameters. The heatmap at the top corresponds to the GGNN complex with STG WT, whereas that at the bottom corresponds to the mutated version. Compared to the STG WT system, the number of HBs formed and their occupancy were barely affected by the STG V143L mutation.


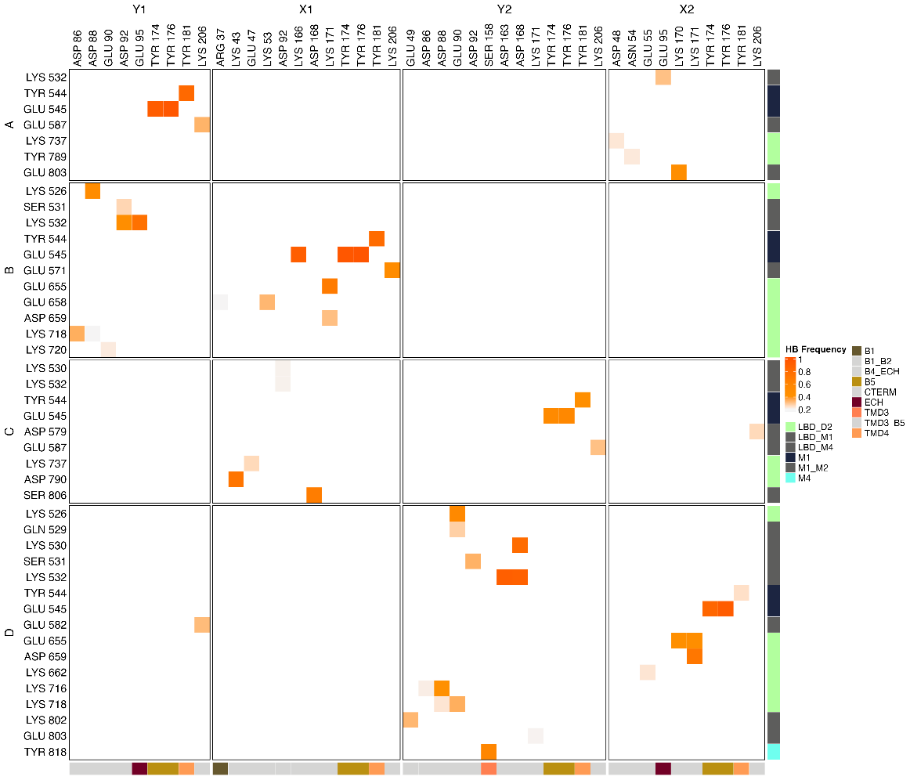

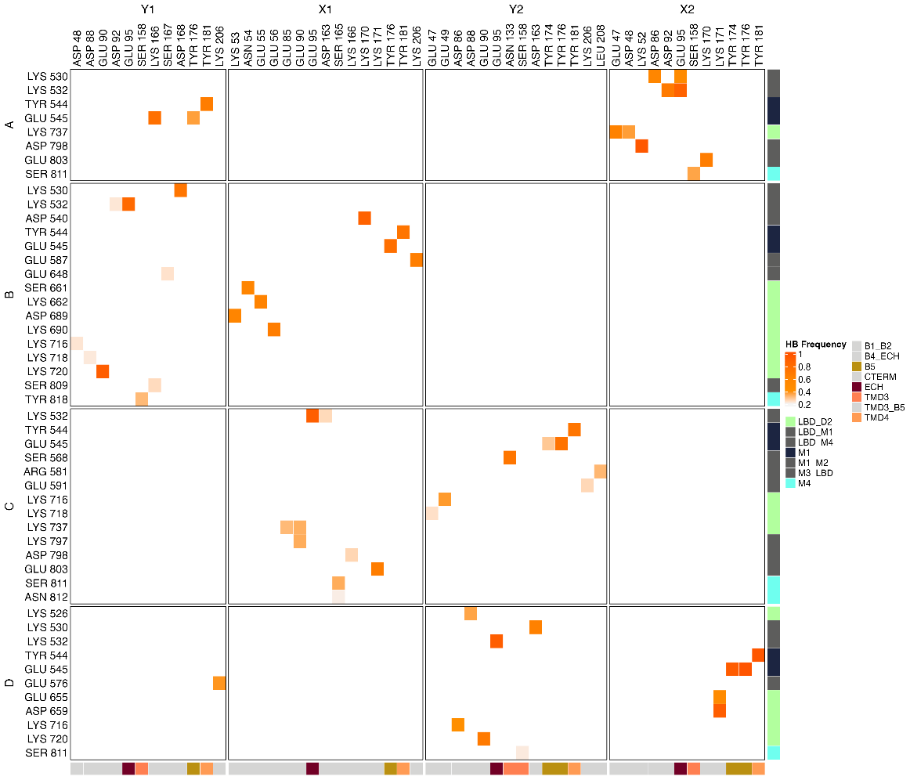


**Figure SI 7. Hydrogen bond frequency of complex GGGN** **per substructure.** The HB frequency was calculated using the Python package GetContacts with default parameters. The heatmap at the top corresponds to the GGGN complex with STG WT, whereas that at the bottom corresponds to the mutated version. Compared with the STG WT system, the number of HBs formed and their occupancy increased in the presence of the STG V143L mutation.


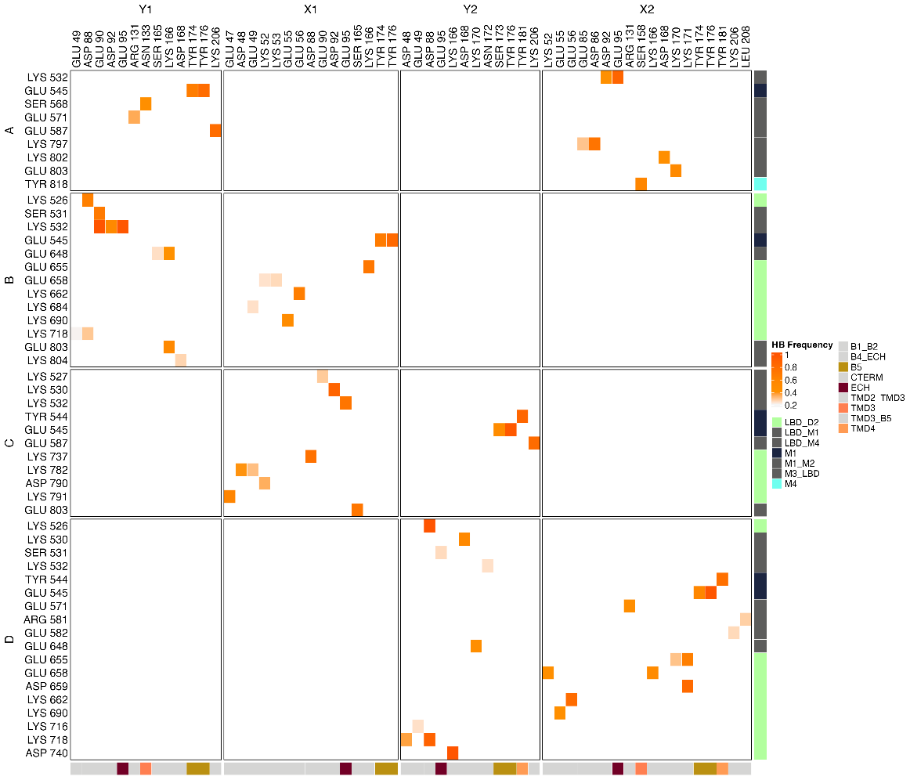

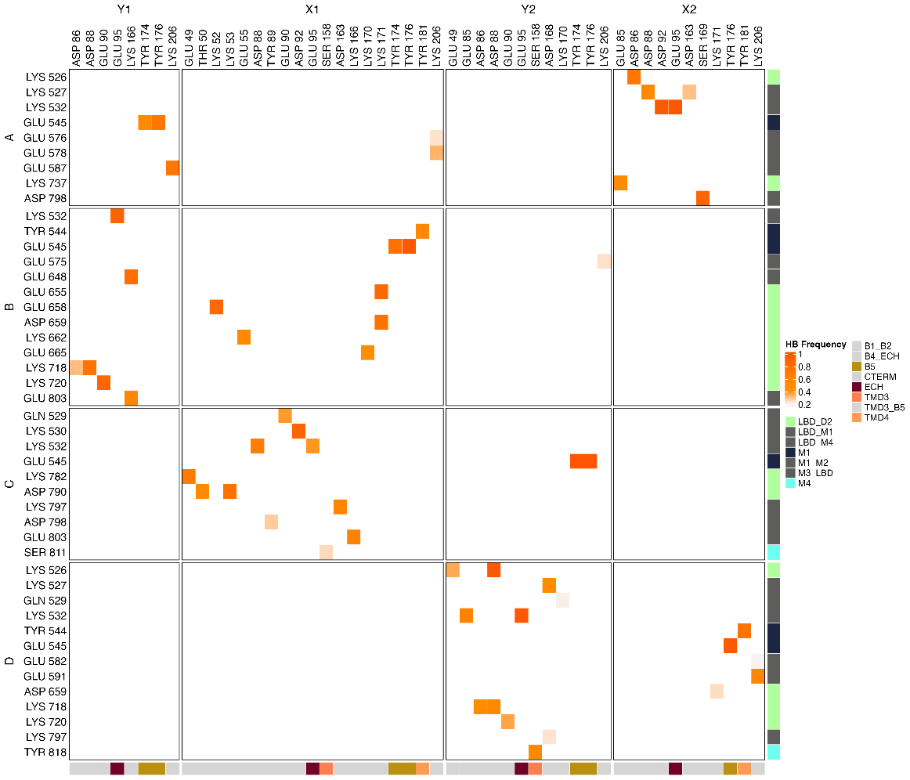


Figure SI 8. Hydrogen bond frequency of complex GNGN1 per substructure. The HB frequency was calculated using the Python package GetContacts with default parameters. The heatmap at the top corresponds to the GNGN1 complex with STG WT, whereas that at the bottom corresponds to the mutated version. Compared with the STG WT system, the number of HBs formed did not increase in the presence of the STG V143L mutation, whereas their occupancy tended to increase.


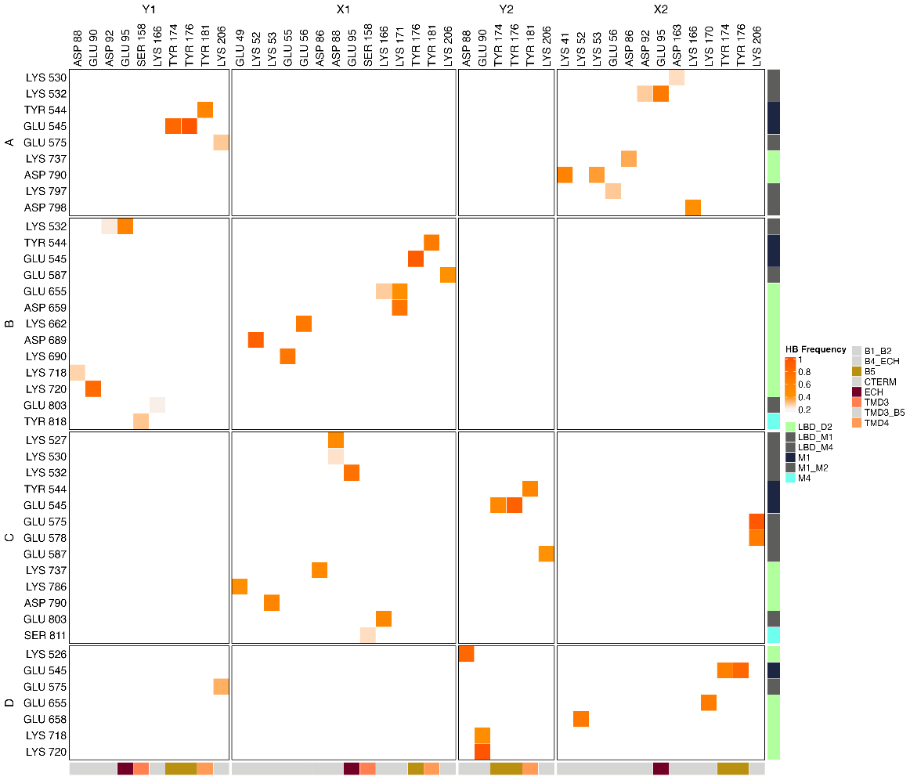

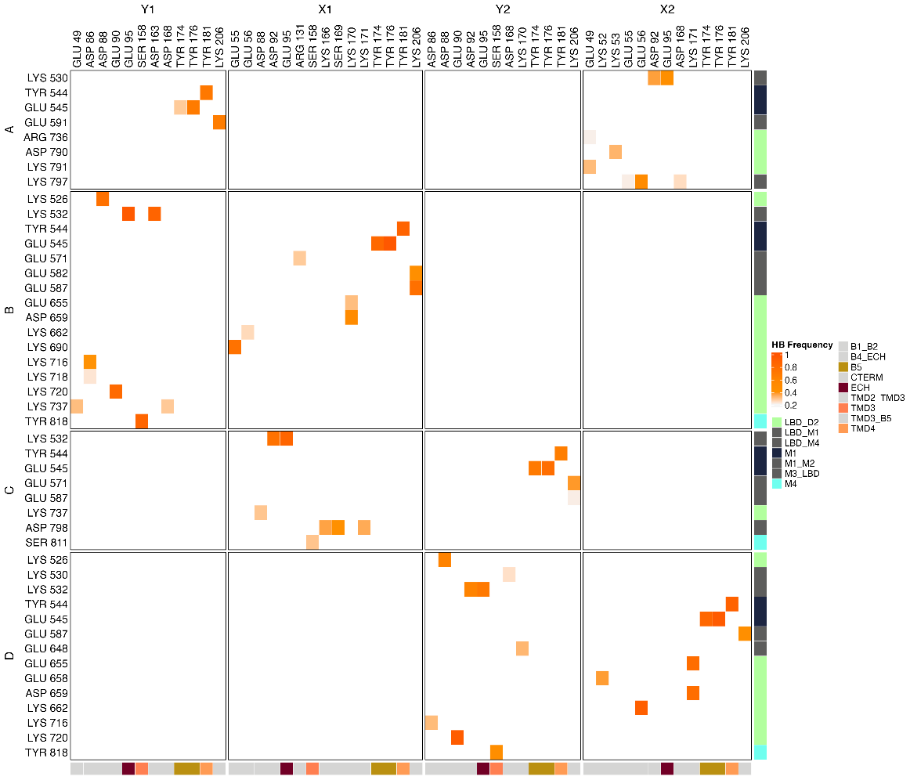


**Figure SI 9. Hydrogen bond frequency of complex GNGN2** **per substructure.** The HB frequency was calculated using the Python package GetContacts with default parameters. The heatmap at the top corresponds to the GNGN2 complex with STG WT, whereas that at the bottom corresponds to the mutated version. Compared with the STG WT system, the number of HBs formed and their occupancy increased in the presence of the STG V143L mutation.


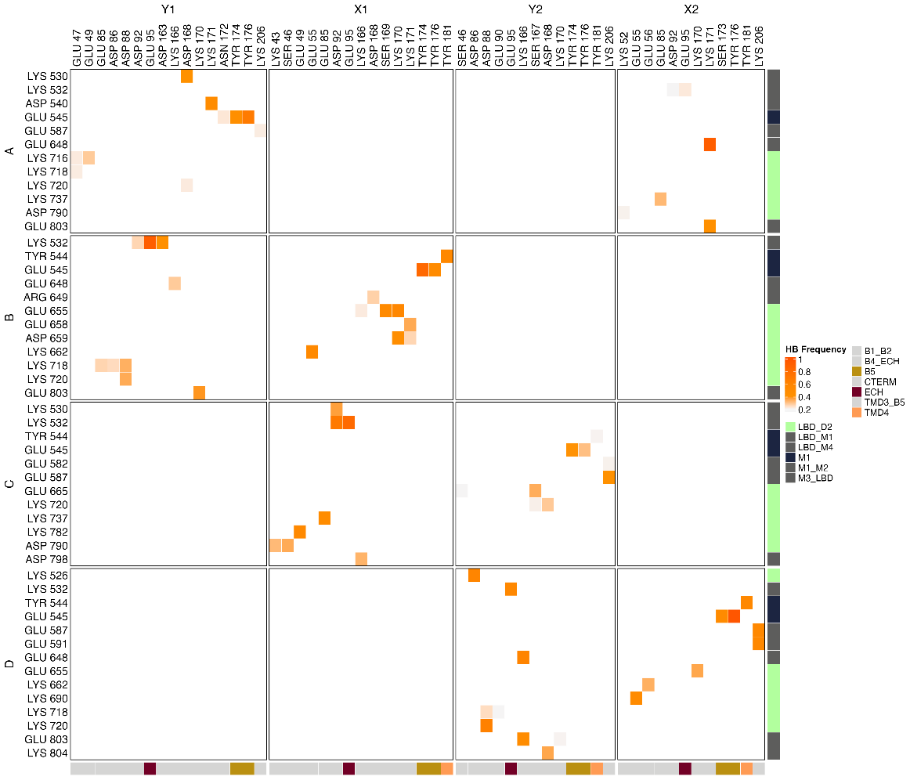

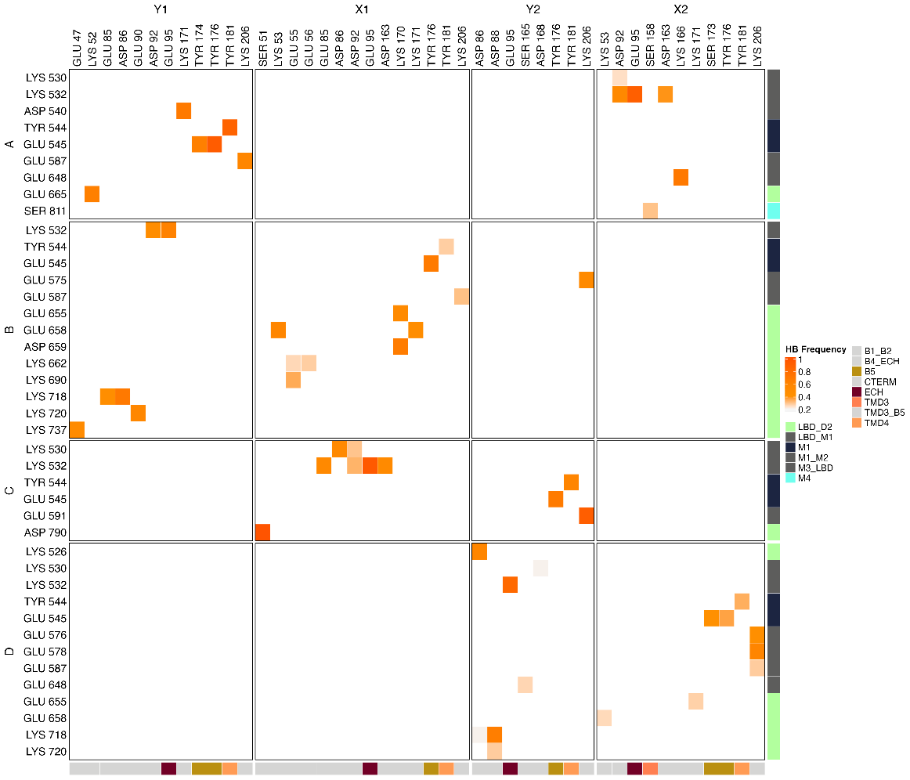


**Figure SI 10. Hydrogen bond frequency of complex GGGG** **per substructure.** The HB frequency was calculated using the Python package GetContacts with default parameters. The heatmap at the top corresponds to the GGGG complex with STG WT, whereas that at the bottom corresponds to the mutated version. Compared with the STG WT system, the number of HBs formed and their occupancy increased in the presence of the STG V143L mutation.


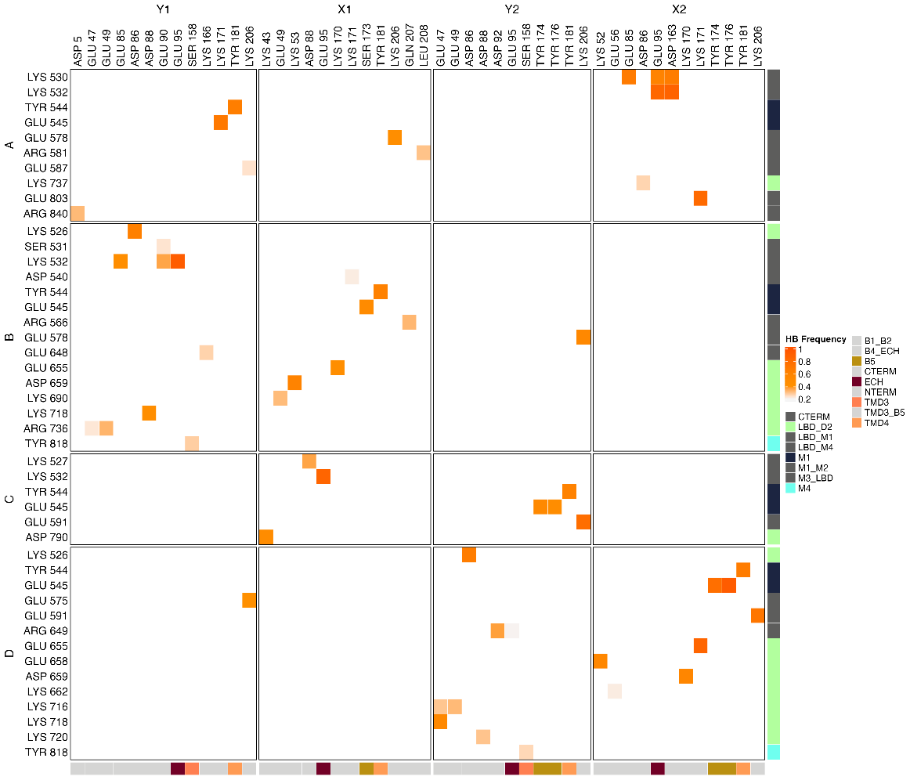

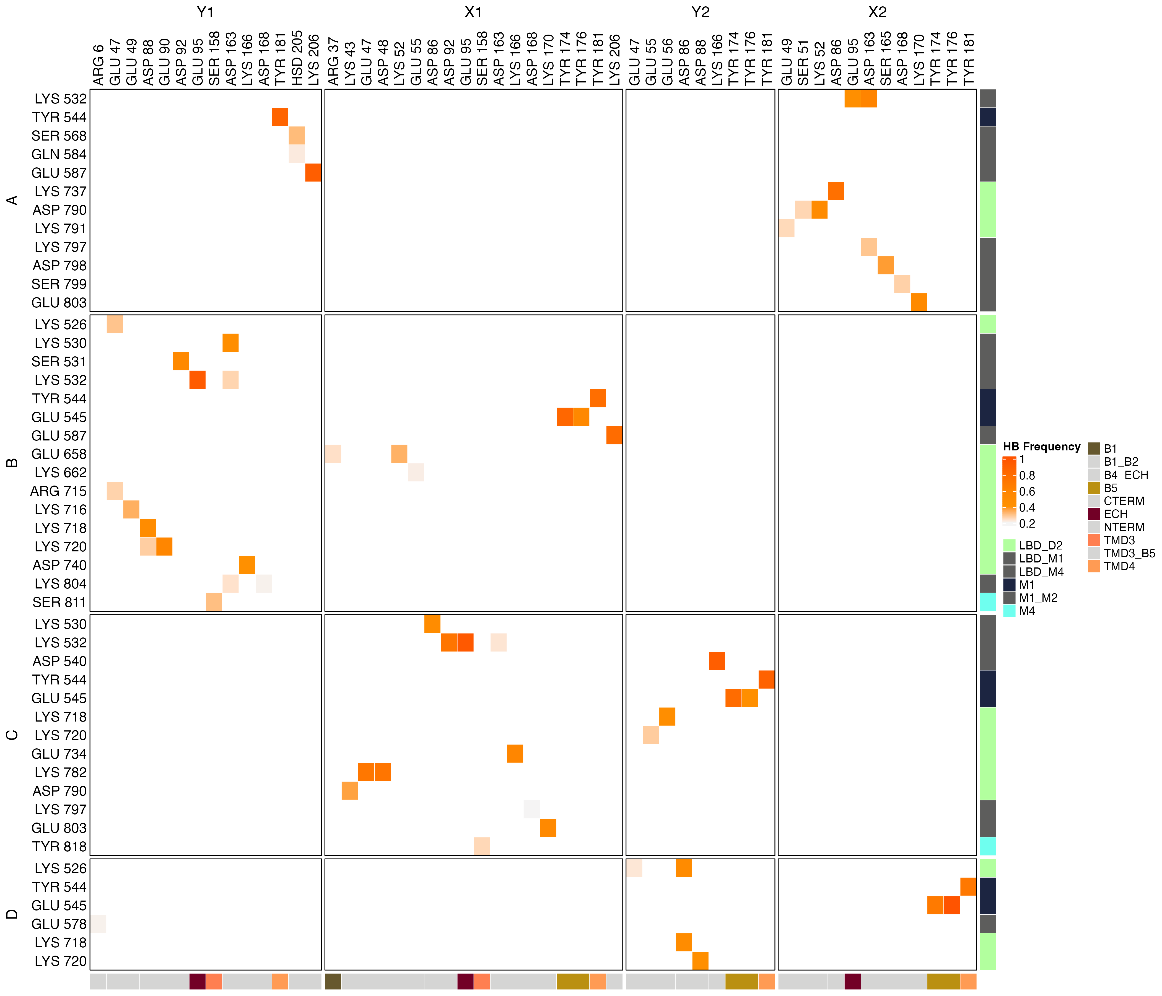


**Figure SI 11. Hydrogen bond frequency of complex 5WEO** **per substructure.** The HB frequency was calculated using the Python package GetContacts with default parameters. The heatmap at the top corresponds to the 5WEO complex with STG WT, whereas that at the bottom corresponds to the mutated version. Compared with the STG WT system, the number of HBs formed and their occupancy increased in the presence of the STG V143L mutation.


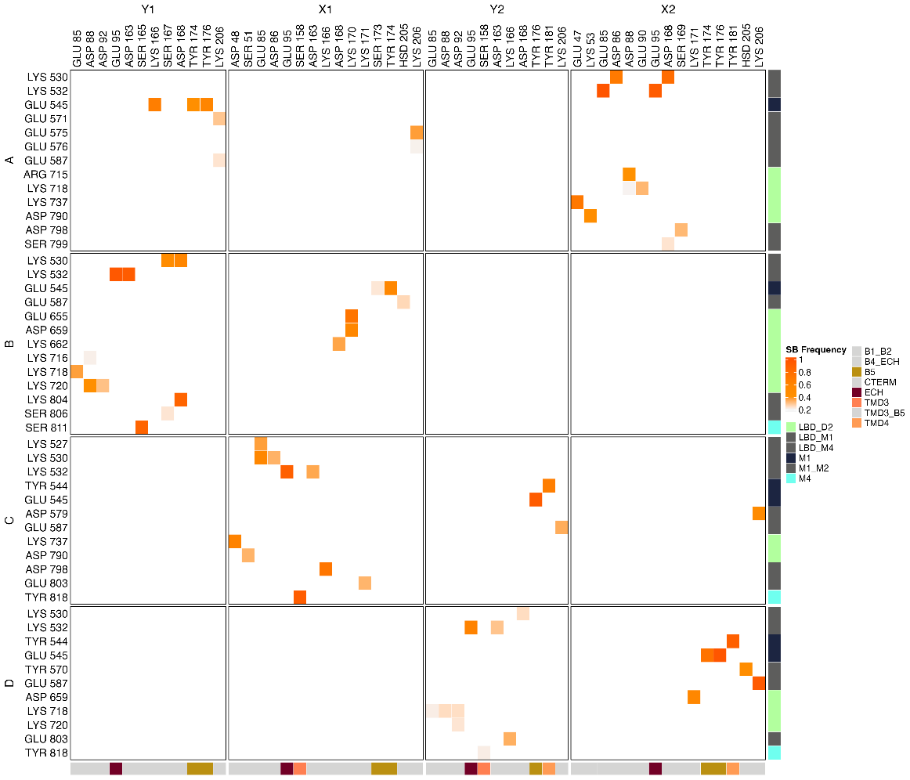

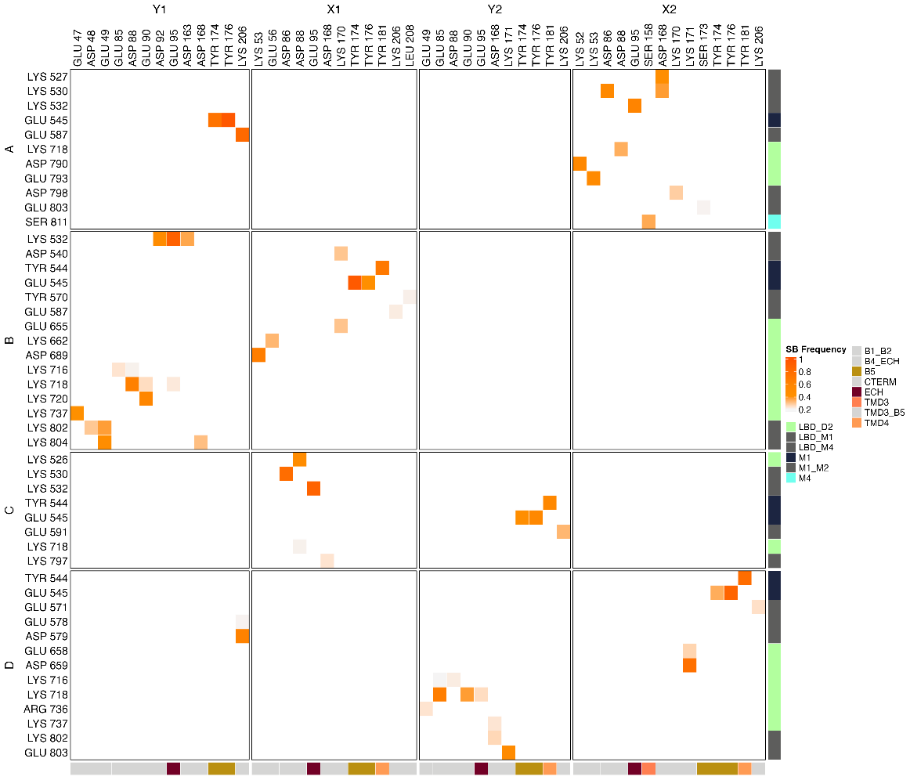


Figure SI 12. Salt bridge frequency of complex NNNN per substructure. The SB frequency was calculated using the Python package GetContacts with default parameters. The heatmap at the top corresponds to the NNNN complex with STG WT, whereas that at the bottom corresponds to the mutated version. Compared with the STG WT system, the number of SBs formed, and their occupancy decreased in the presence of the STG V143L mutation.


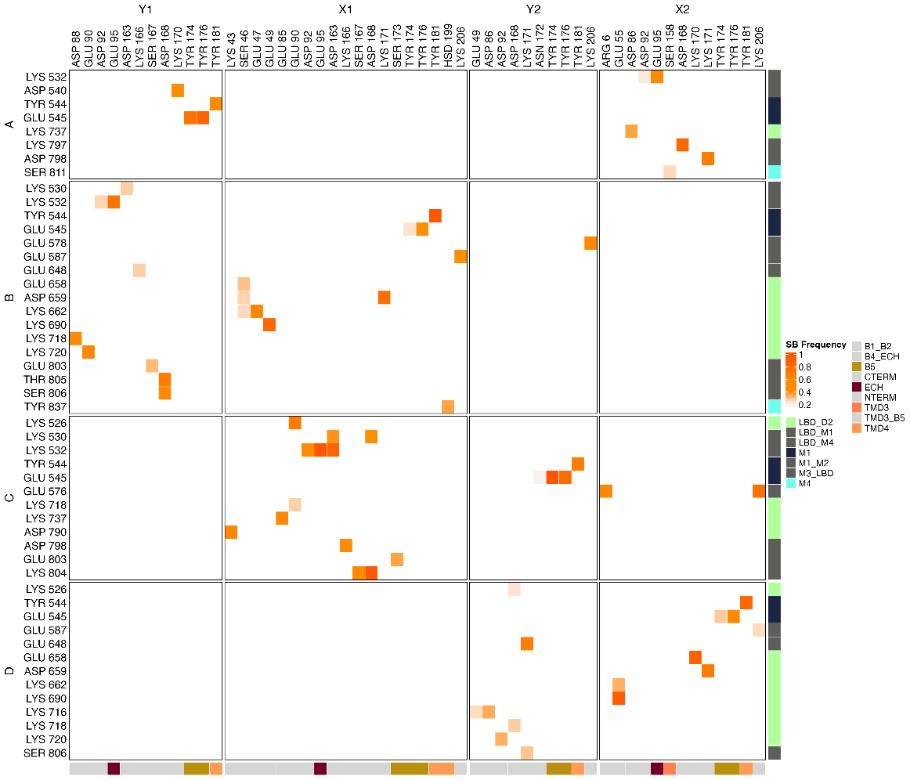

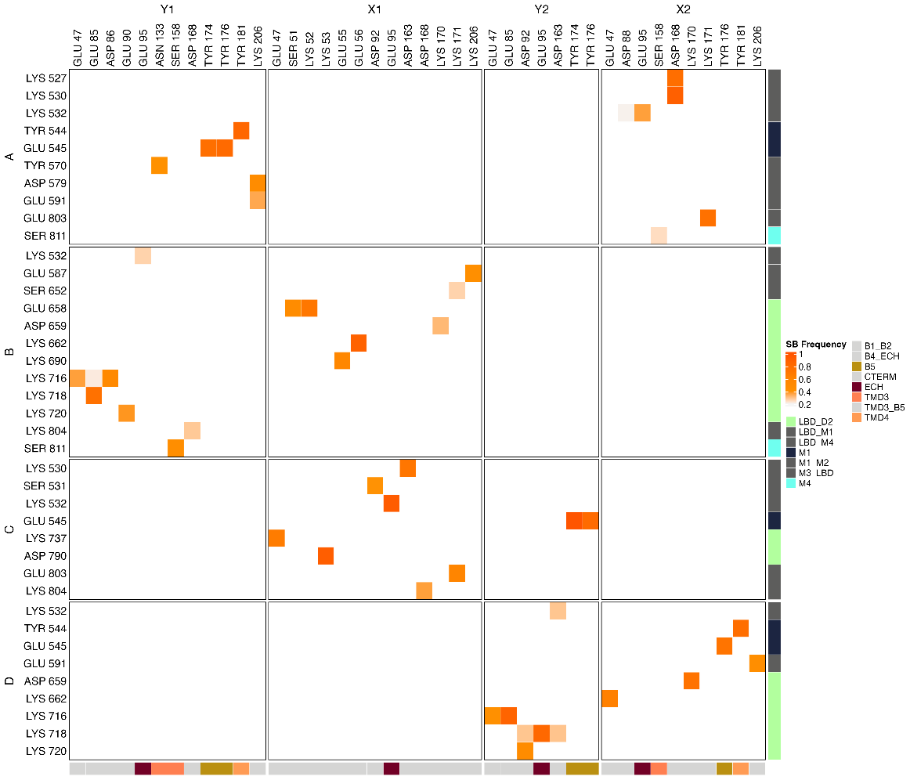


**Figure SI 13. Salt bridge frequency of complex GNNN** **per substructure.** The SB frequency was calculated using the Python package GetContacts with default parameters. The heatmap at the top corresponds to the GNNN complex with STG WT, whereas that at the bottom corresponds to the mutated version. Compared with the STG WT system, the number of SBs formed, and their occupancy decreased in the presence of the STG V143L mutation.


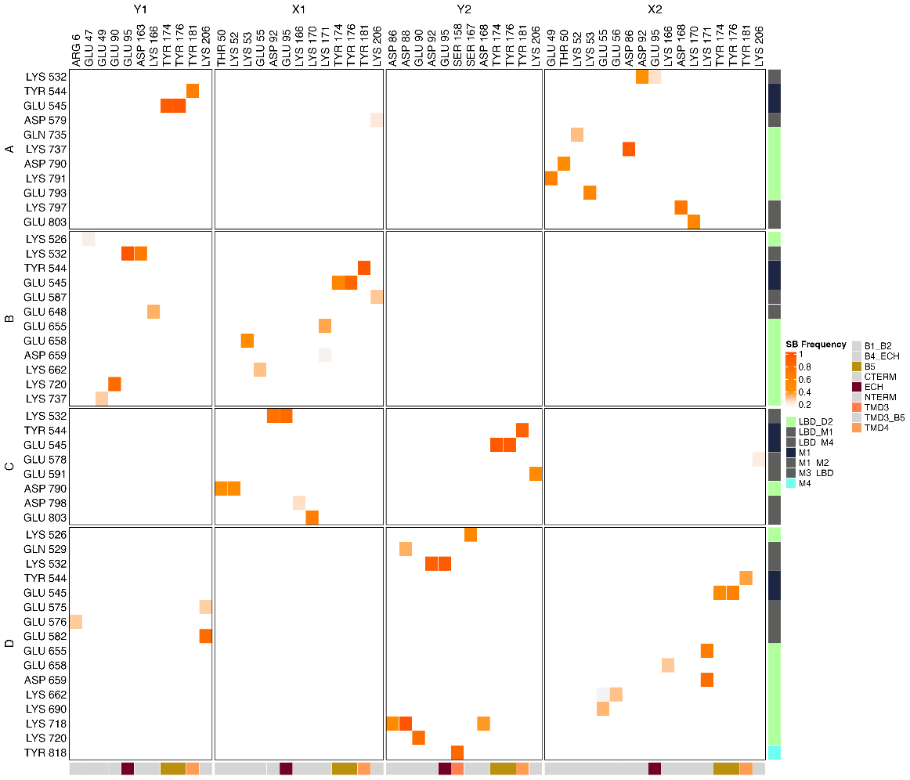

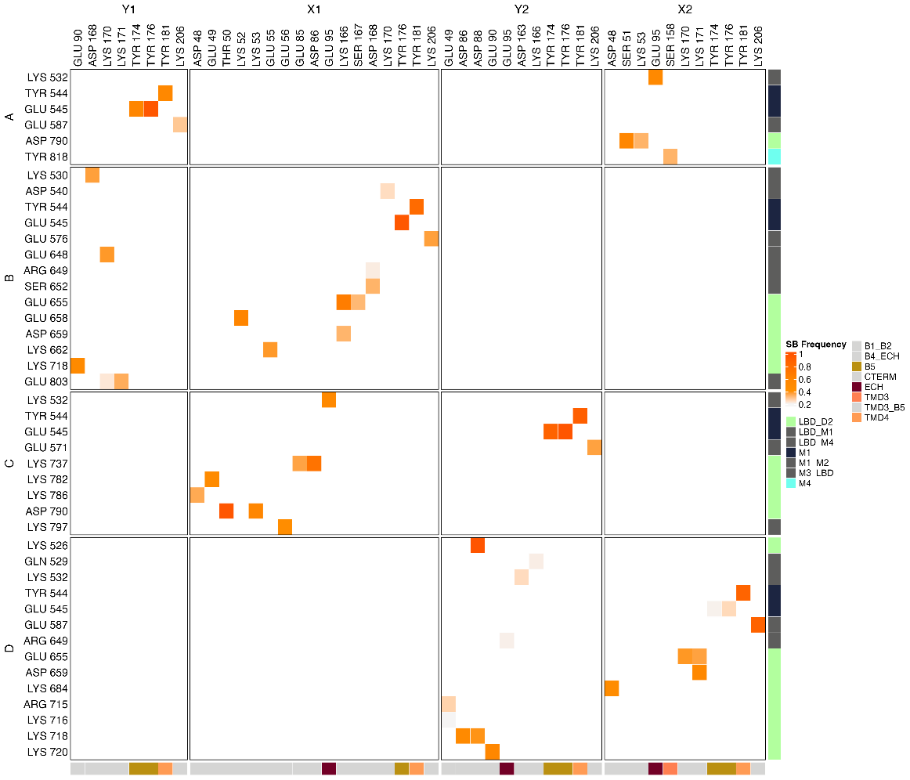


Figure SI 14. Salt-bridge frequency of complex GGNN per substructure. The SB frequency was calculated using the Python package GetContacts with default parameters. The heatmap at the top corresponds to the GGNN complex with STG WT, whereas that at the bottom corresponds to the mutated version. Compared with the STG WT system, the number of SBs formed, and their occupancy decreased in the presence of the STG V143L mutation.


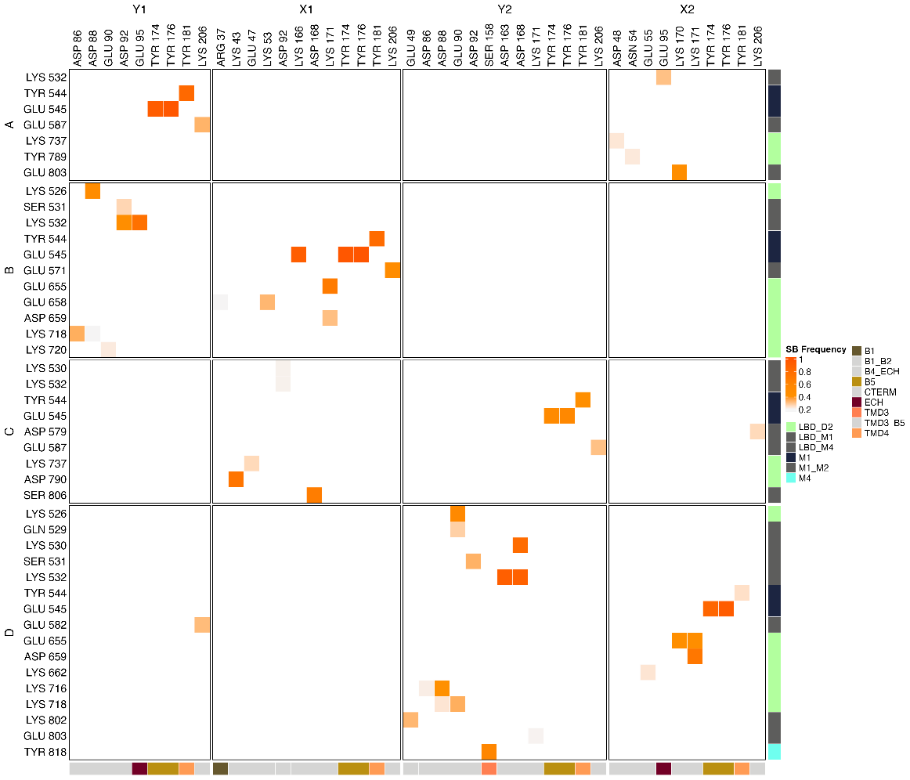

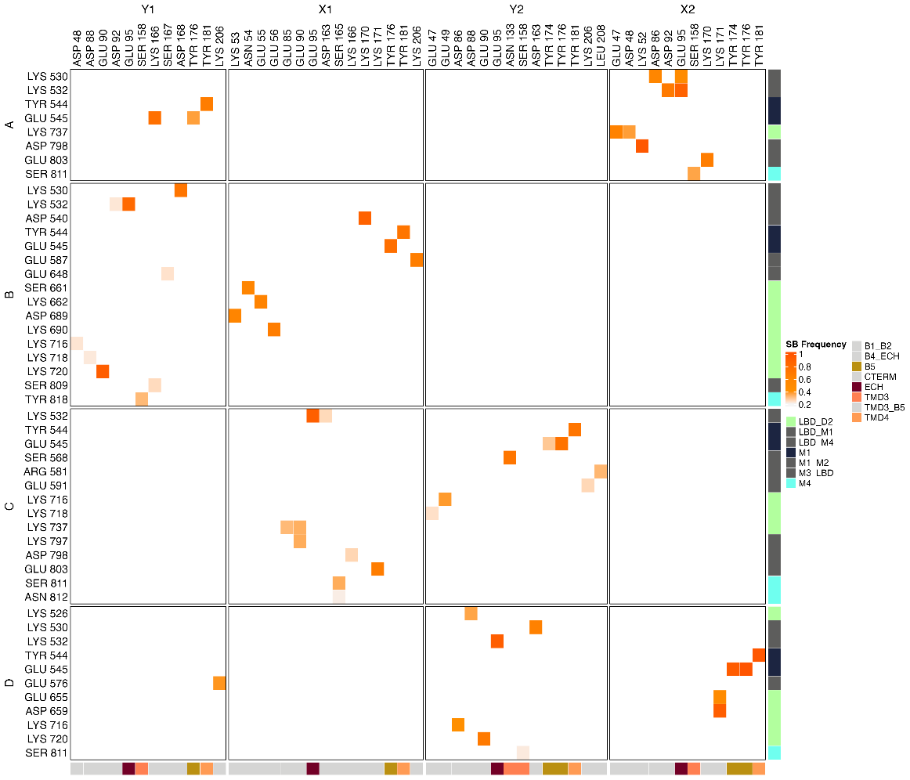


**Figure SI 15. Salt-bridge frequency of complex GGGN** **per substructure.** The SB frequency was calculated using the Python package GetContacts with default parameters. The heatmap at the top corresponds to the GGGN complex with STG WT, whereas that at the bottom corresponds to the mutated version. Compared with the STG WT system, the number of SBs formed, and their occupancy increased in the presence of the STG V143L mutation.


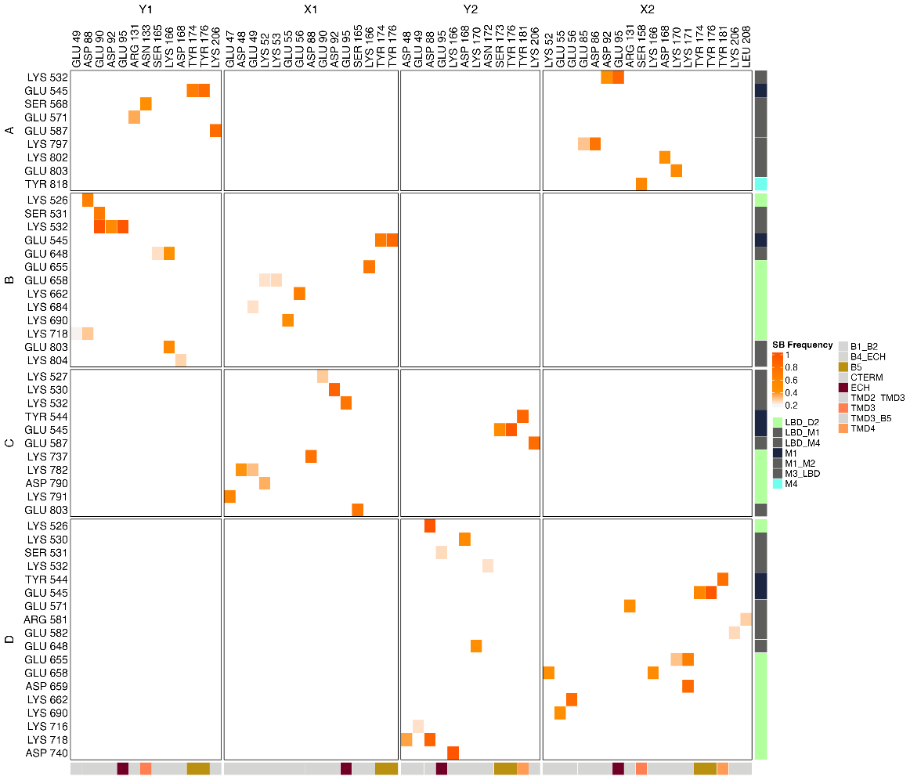

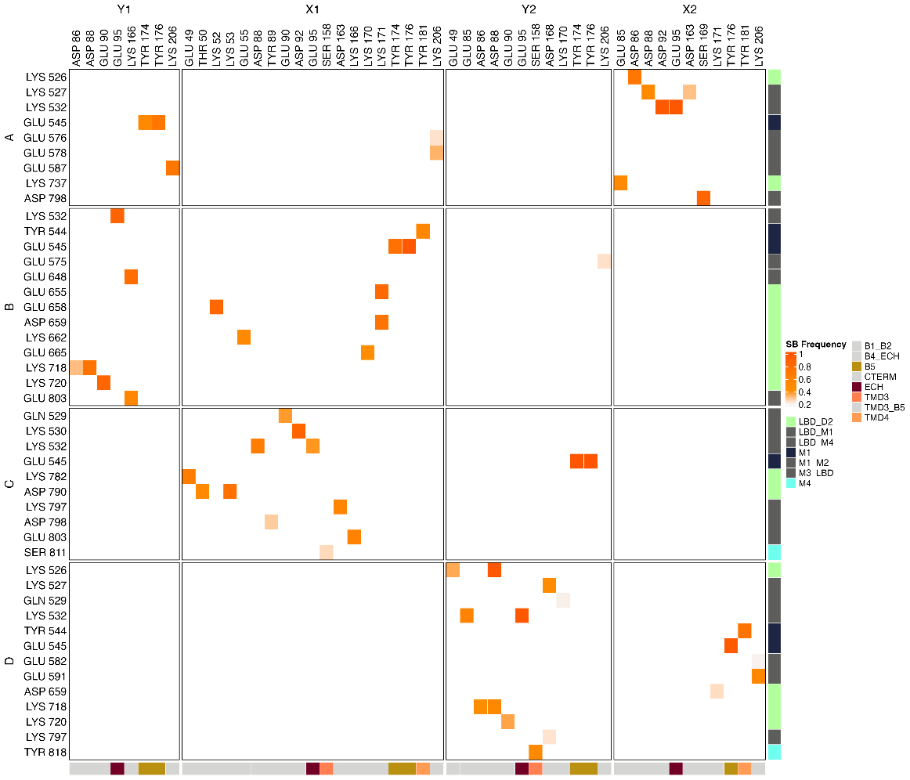


Figure SI 16. Salt bridge frequency of complex GNGN1 per substructure. The SB frequency was calculated using the Python package GetContacts with default parameters. The heatmap at the top corresponds to the GNGN1 complex with STG WT, whereas that at the bottom corresponds to the mutated version. Compared to the STG WT system, the number of SBs formed decreased in the presence of the STG V143L mutation, whereas their occupancy increased.


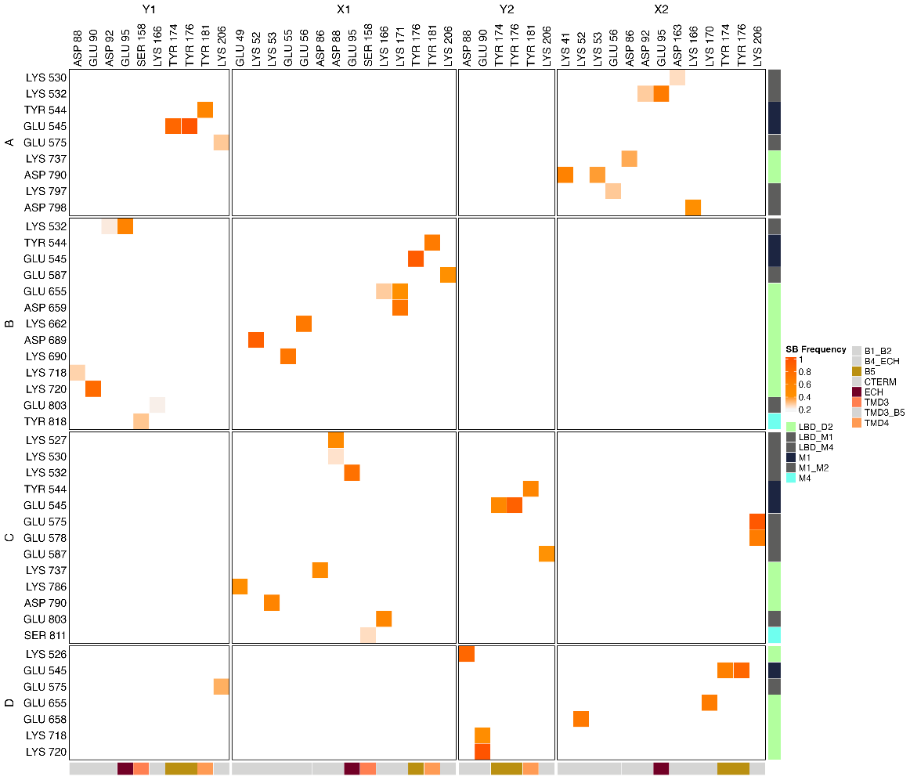

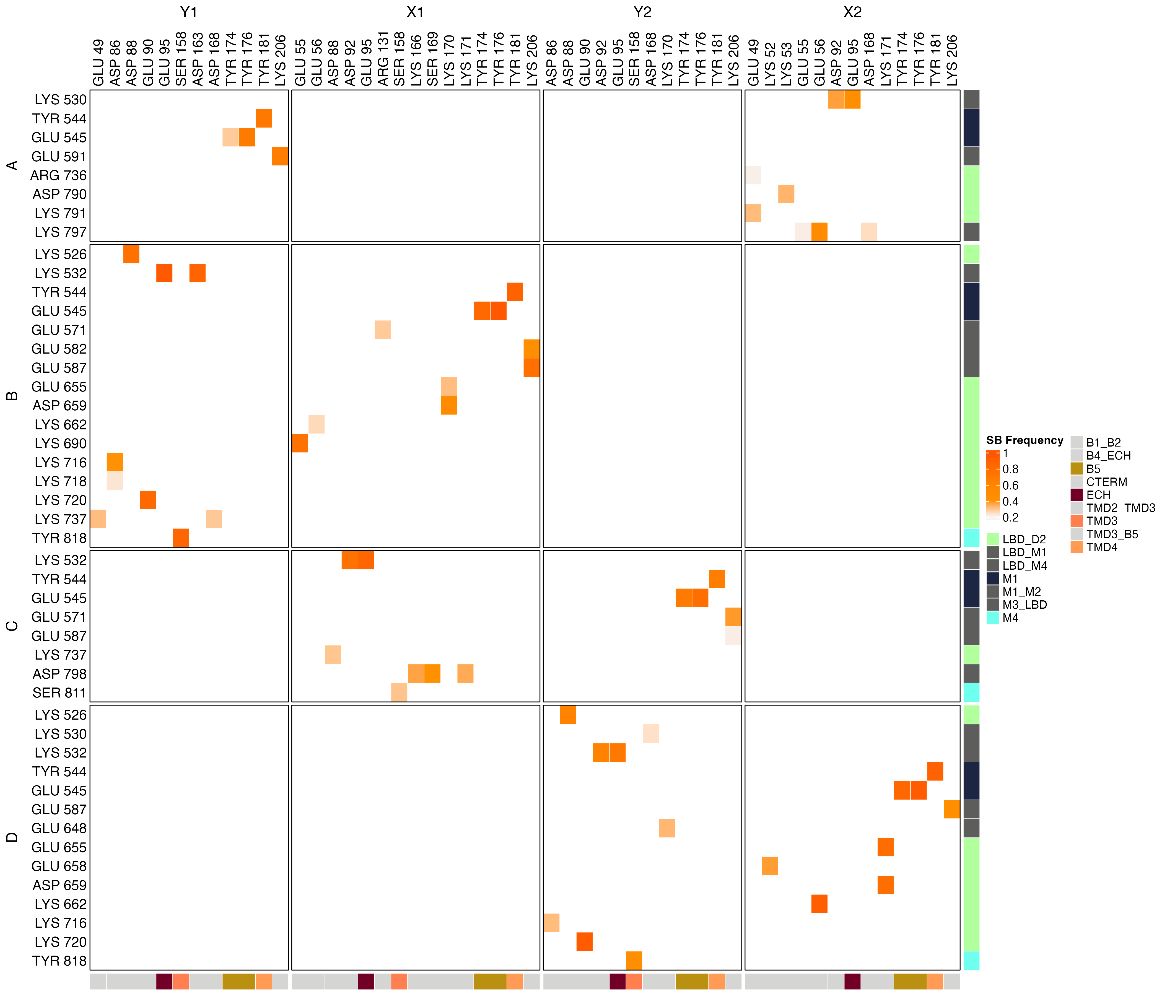


**Figure SI 17. Salt bridge frequency of complex GNGN2** **per substructure.** The SB frequency was calculated using the Python package GetContacts with default parameters. The heatmap at the top corresponds to the GNGN2 complex with STG WT, whereas that at the bottom corresponds to the mutated version. Compared with the STG WT system, the number of SBs formed, and their occupancy increased in the presence of the STG V143L mutation.


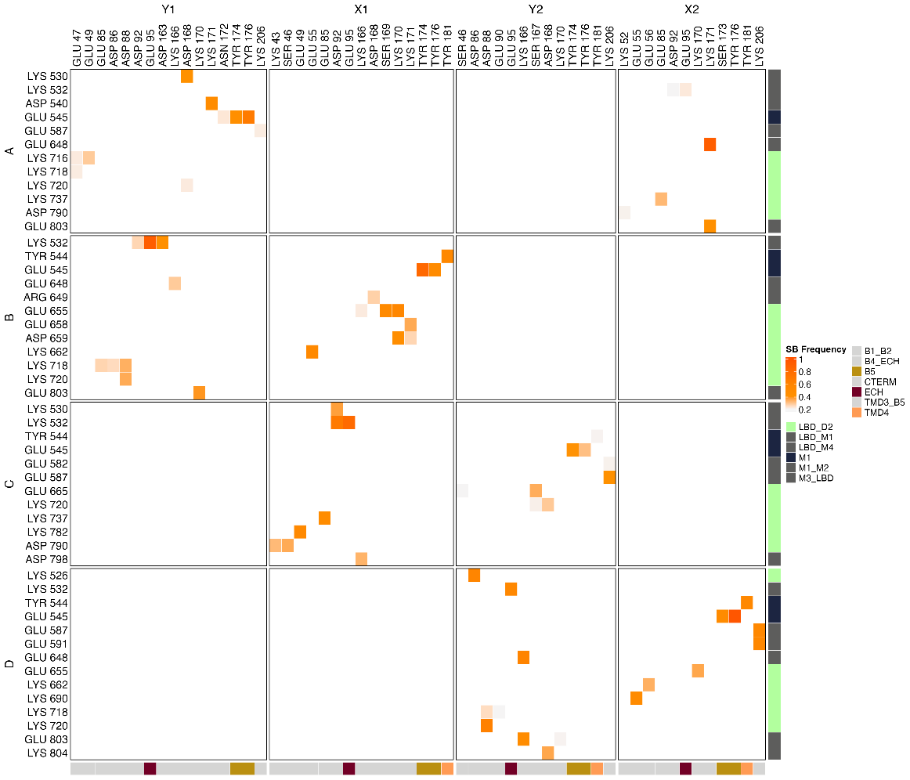

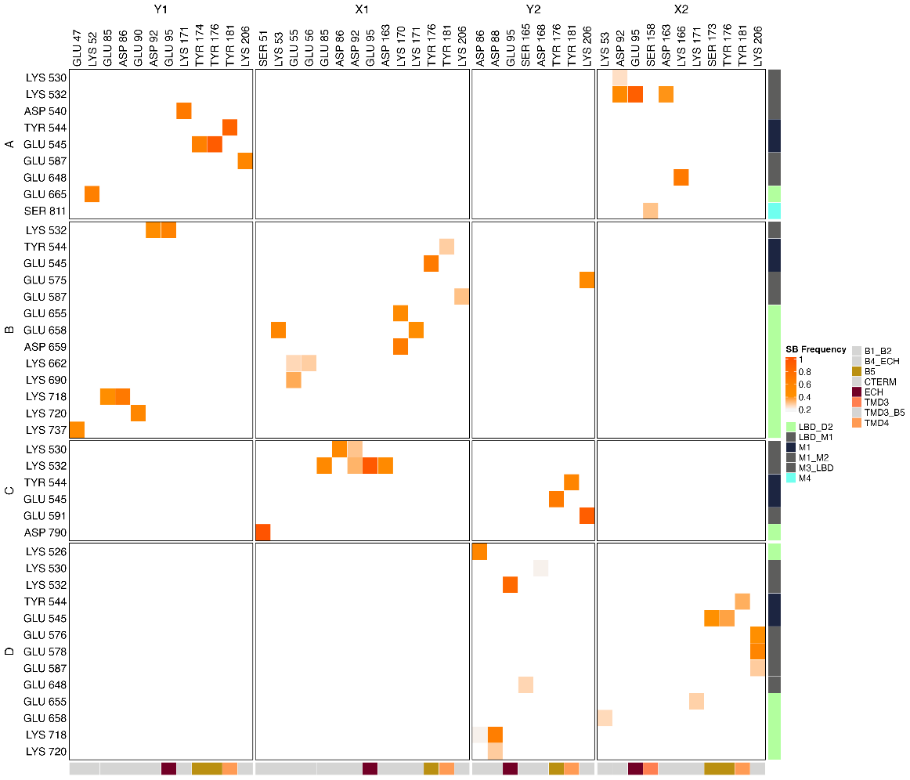


Figure SI 18. Salt bridge frequency of complex GGGG per substructure. The SB frequency was calculated using the Python package GetContacts with default parameters. The heatmap at the top corresponds to the GGGG complex with STG WT, whereas that at the bottom corresponds to the mutated version. Compared with the STG WT system, the number of SBs formed, and their occupancy decreased in the presence of the STG V143L mutation.


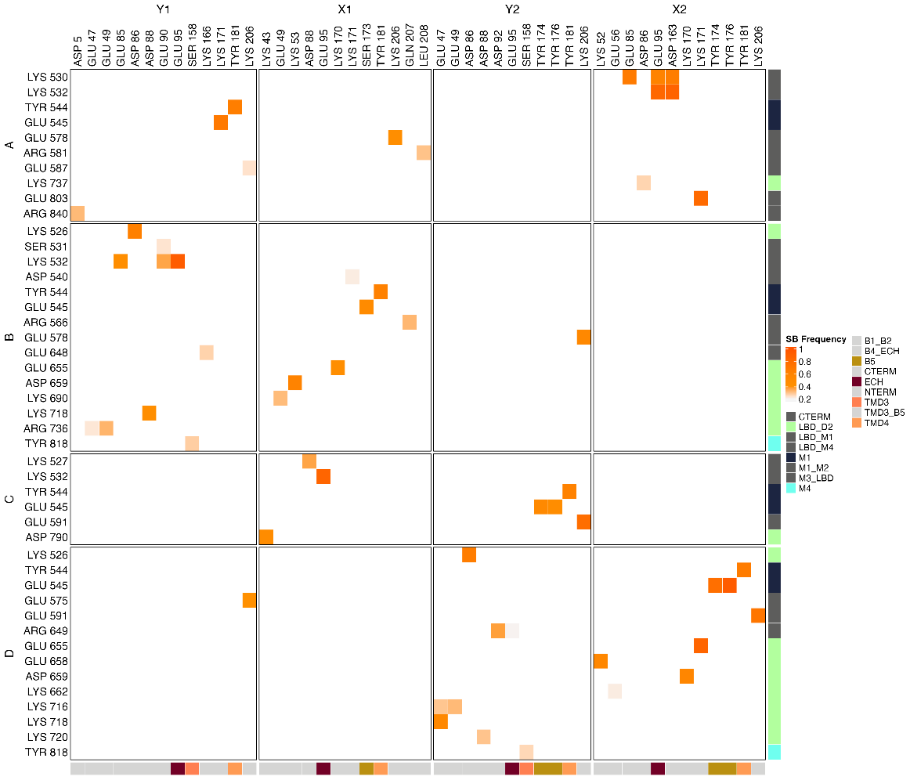

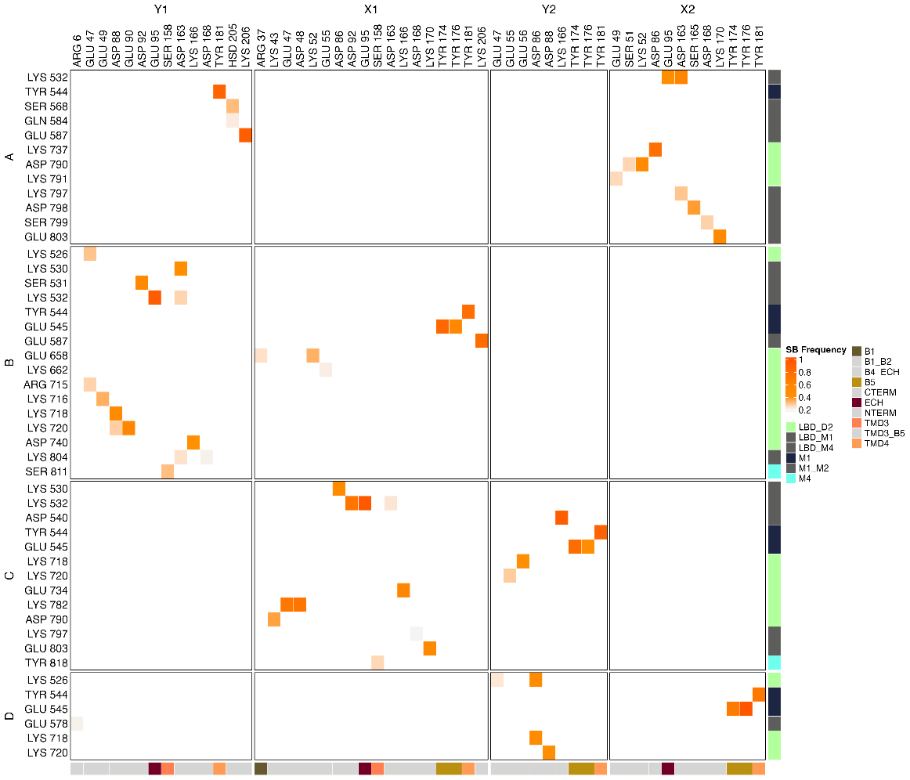


**Figure SI 19. Salt bridge frequency of complex 5WEO** **per substructure.** The SB frequency was calculated using the Python package GetContacts with default parameters. The heatmap at the top corresponds to the 5WEO complex with STG WT, whereas that at the bottom corresponds to the mutated version. Compared with the STG WT system, the number of SBs formed, and their occupancy decreased in the presence of the STG V143L mutation.


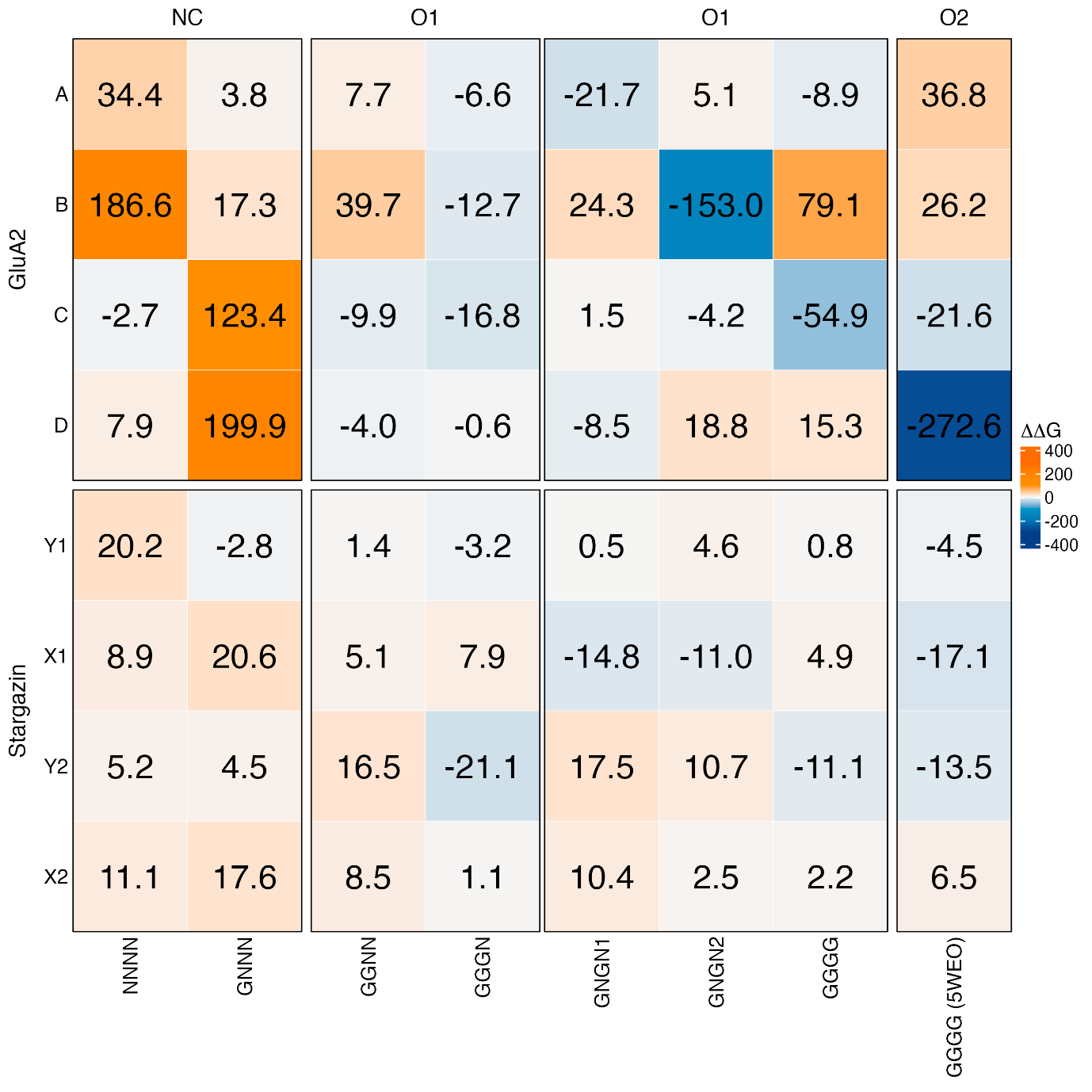


Figure SI 20. ∆∆GB per structure. The values were expressed in kcal/mol and were calculated by subtracting the values of ΔG_V143L_ from those of ΔG_WT_. The ΔG values were calculated by subtracting the G_AMPAR:STG complex_ G_GluA2_ and G_STG_, which were performed using the AMBER Molecular Mechanics Poisson Boltzmann Surface Area method (MMPBSA), as implemented in the gmx_MMPBSA package. The STG subunits did not have large ∆∆G values; however, the STG WT proteins in the NC structures tended to be more stable, and those in the O1 and O2 structures tended to be more stable in the STG V143L system. The GluA2 and NC structures tended to be more stable in the STG WT system, especially in chains B, C, and D, whereas the O1 and O2 structures tended to be more stable in the STG V143L system, particularly in chains B of GNGN2 and D of 5WEO.


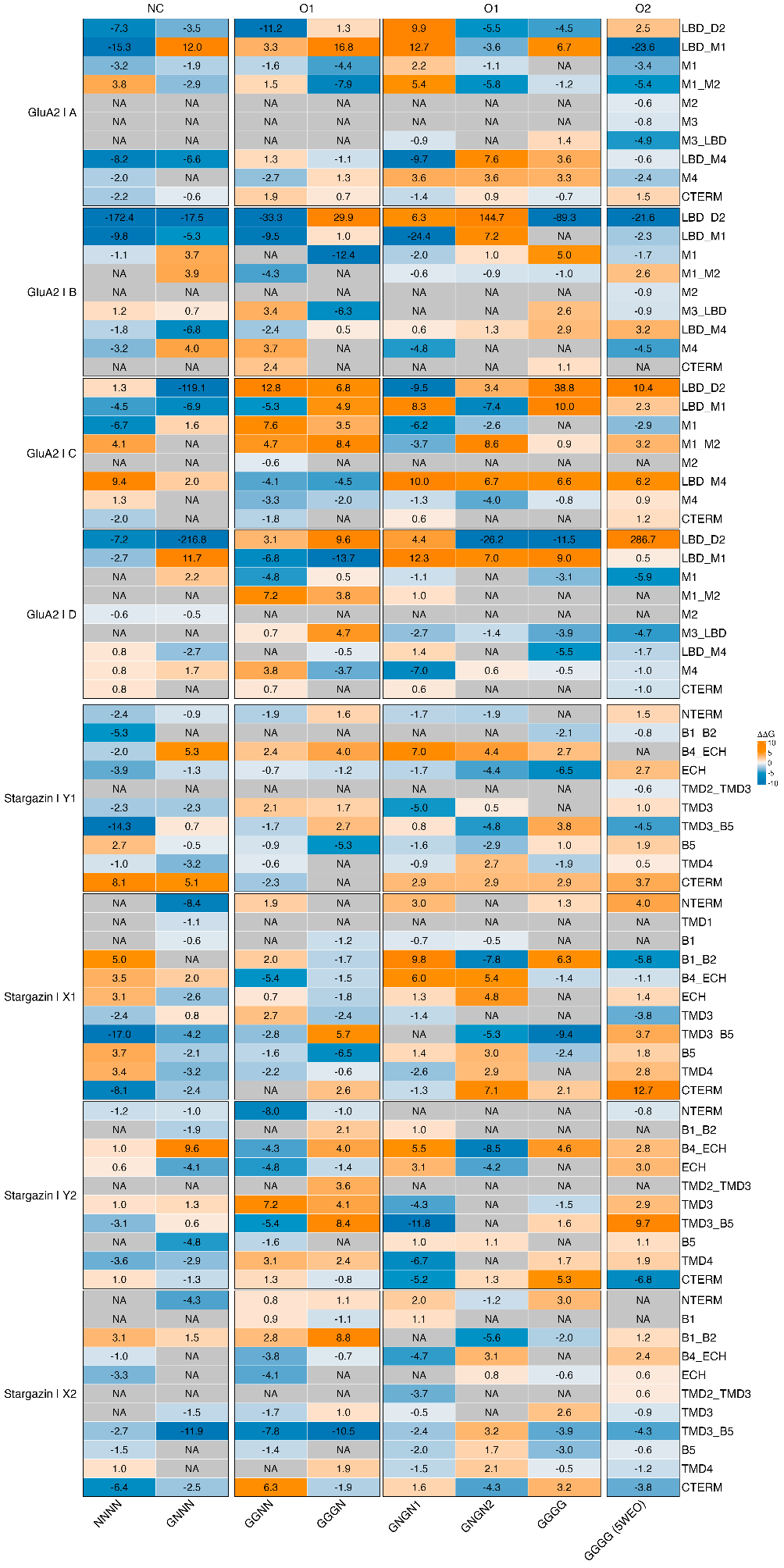


Figure SI 21. ∆G_Binding_ for each substructure. The values were expressed in kcal/mol and were calculated by subtracting the values of ΔG_V143L_ from those of ΔG_WT_. The ΔG values were calculated by subtracting the G_AMPAR:STG_ complex G_GluA2_ and G_STG_, which were performed using the AMBER MMPBSA, as implemented in the gmx_MMPBSA package. In GluA2, the V143L mutation tended to decrease the interaction strength in LBD-D2 in both NC and O2 state structures. In STG substructures, the mutation decreases the stability of ECH and the loop between TMD3 and β5 in the NC and O1 states, while increasing it in the O2 state.

*
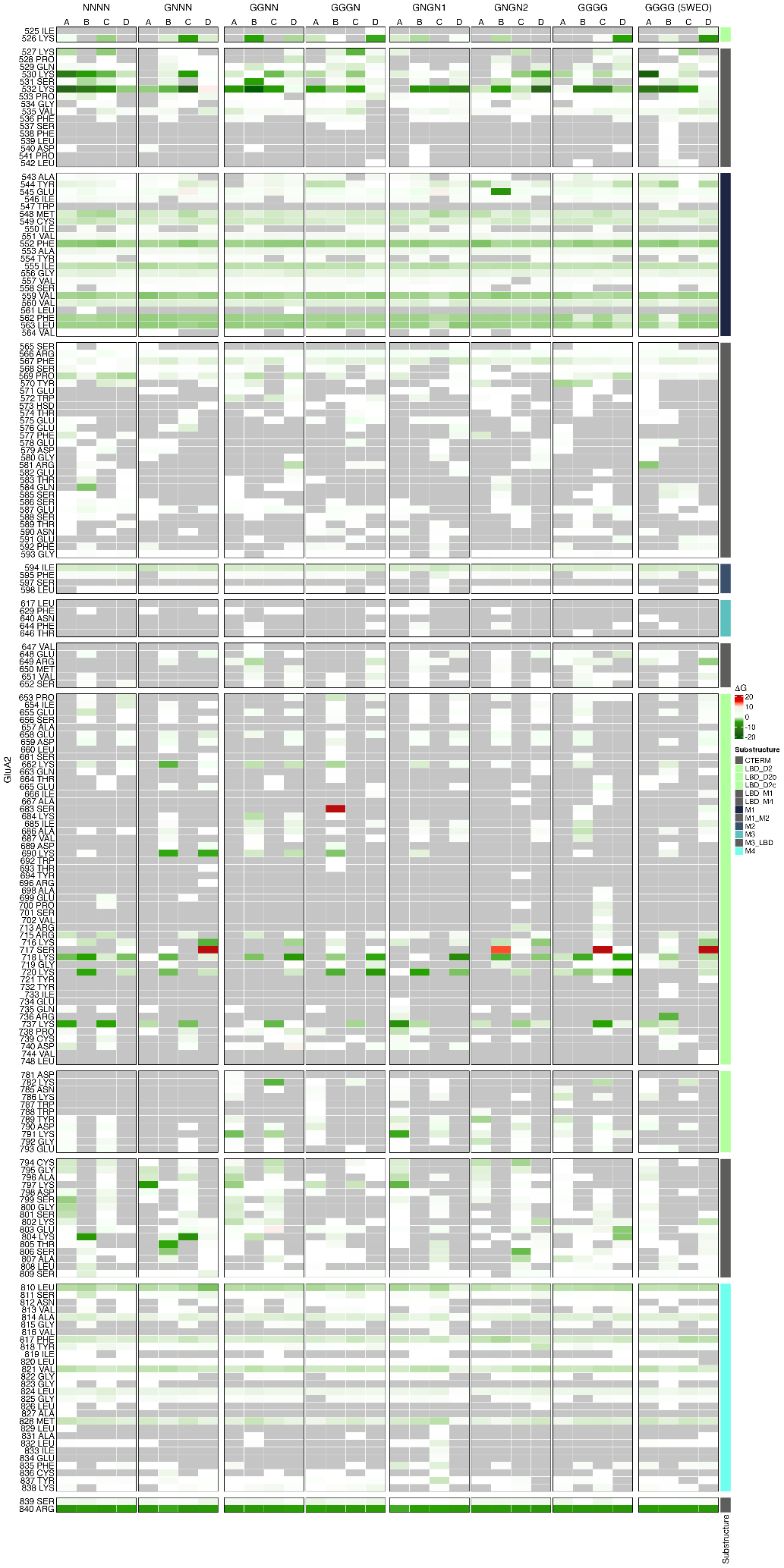
*

**Figure SI 22. ∆G_Binding_ per residue** of GluA2 **in the system with STG WT.** The values are expressed in kcal/mol. The ΔG values were calculated by subtracting the G_AMPAR:STG complex_ G_GluA2_ and G_STG_, which were performed using the AMBER MMPBSA, as implemented in the gmx_MMPBSA package. Residues Glu591 (loop between M1 and M2), Glu655 (2^nd^ domain of LBD), Ser717 (2^nd^ domain of LBD), and Tyr818 (M4) exhibited lower stability in O2 than in O1, whereas Asp798 (loop between LBD and M4) showed increased stability.


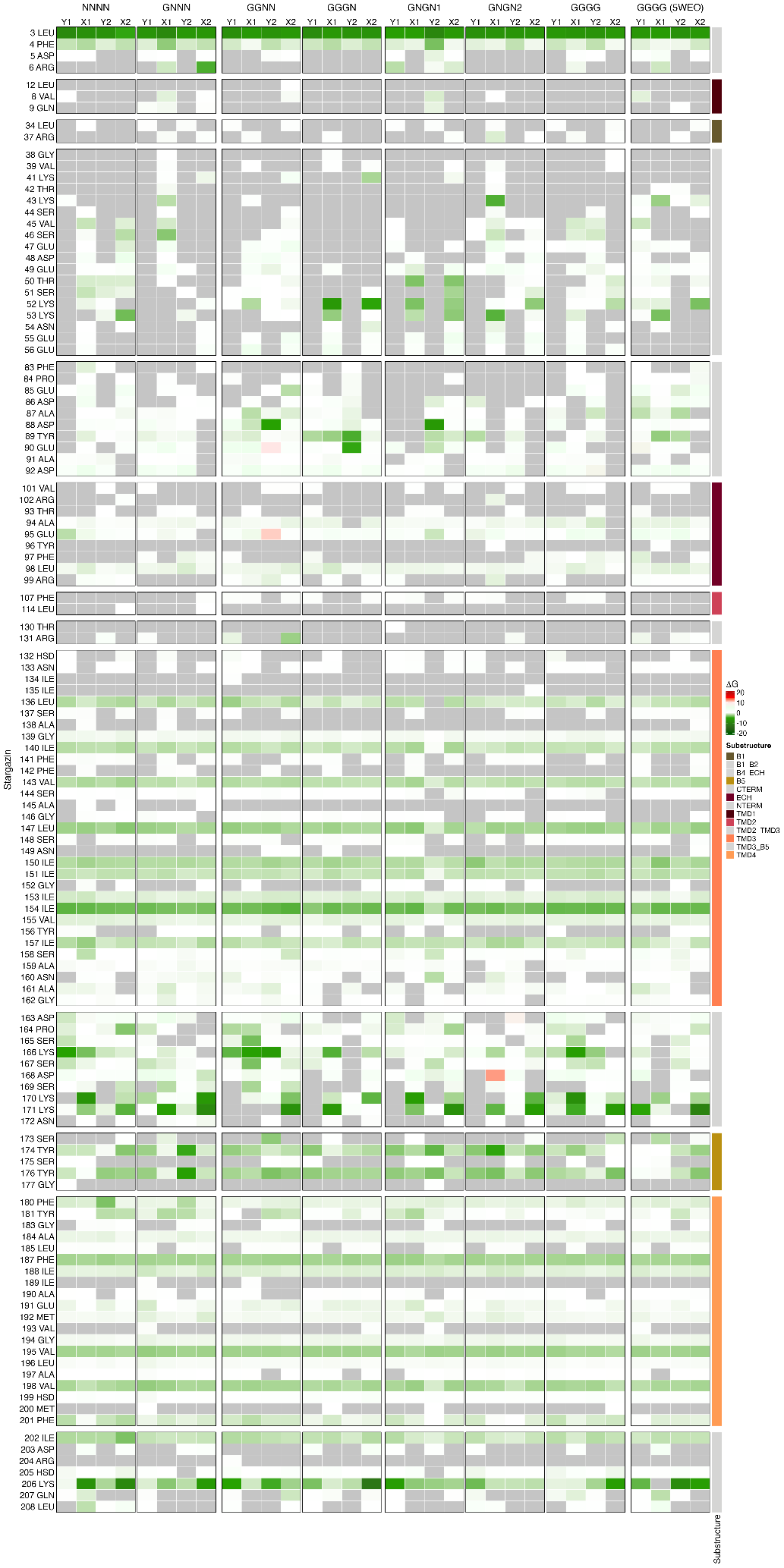


**Figure SI 23. ∆G_Binding_ per residue** of STG **for the system with STG WT.** The values are expressed in kcal/mol. The ΔG values were calculated by subtracting the G_AMPAR:STG_ _complex_ G_GluA2_ and G_STG_, which were performed using the AMBER MMPBSA, as implemented in the gmx_MMPBSA package. Glu95 (ECH) and Ser158 (TMD3) residues were less stable in the O1 and O2 structures.


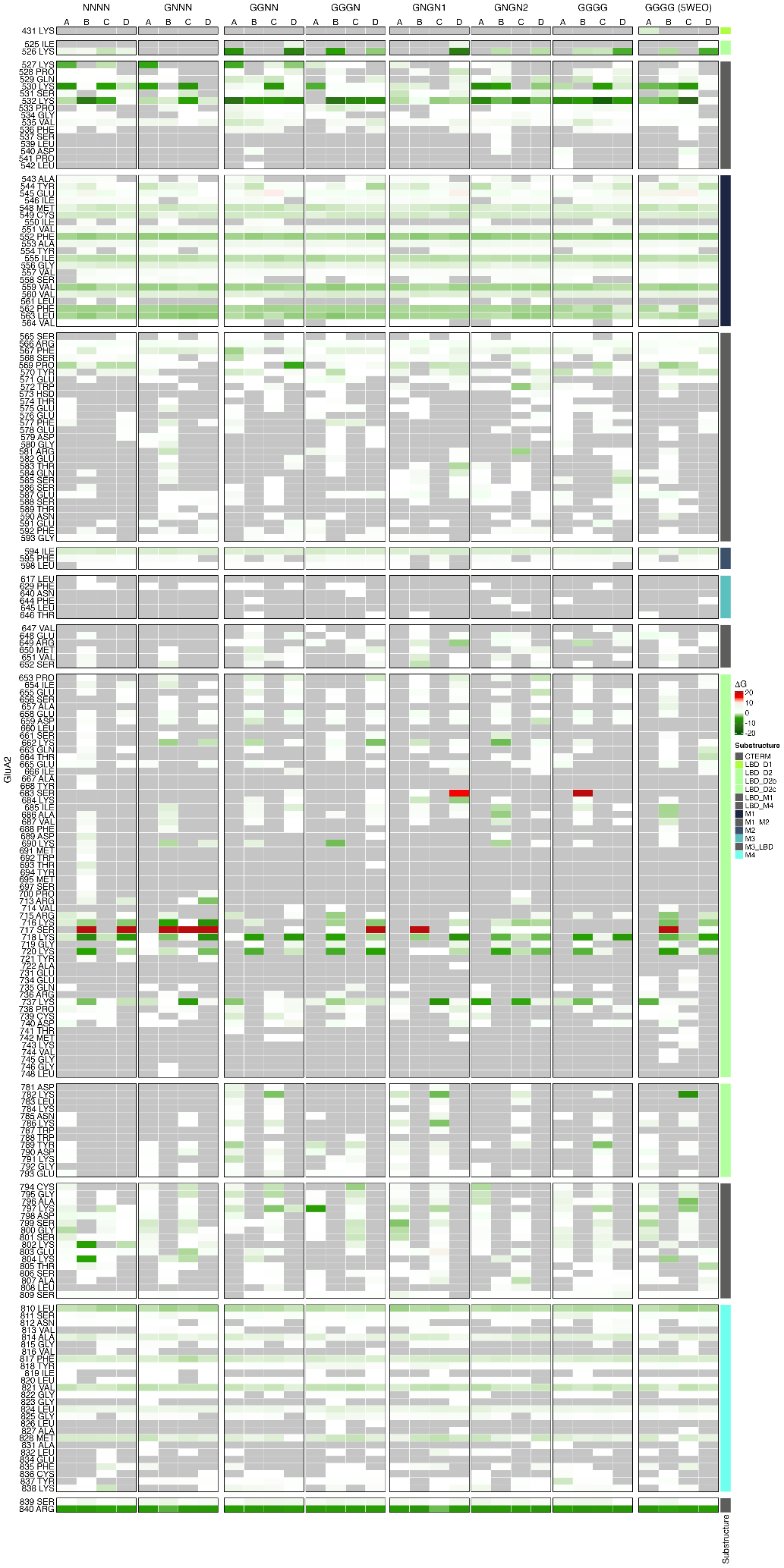


**Figure SI 24. ∆G_Binding_ per GluA2 residue for the system with STG V143L.** The values are expressed in kcal/mol. The ΔG values were calculated by subtracting the G_AMPAR:STG complex_ G_GluA2_ and G_STG_, which were performed using the AMBER MMPBSA, as implemented in the gmx_MMPBSA package. Residues Glu587 (loop between M1 and M2) and Glu648 (loop between M3 and LBD) showed decreased stability in most conductive structures, especially GGGN, GGGG, and 5WEO.


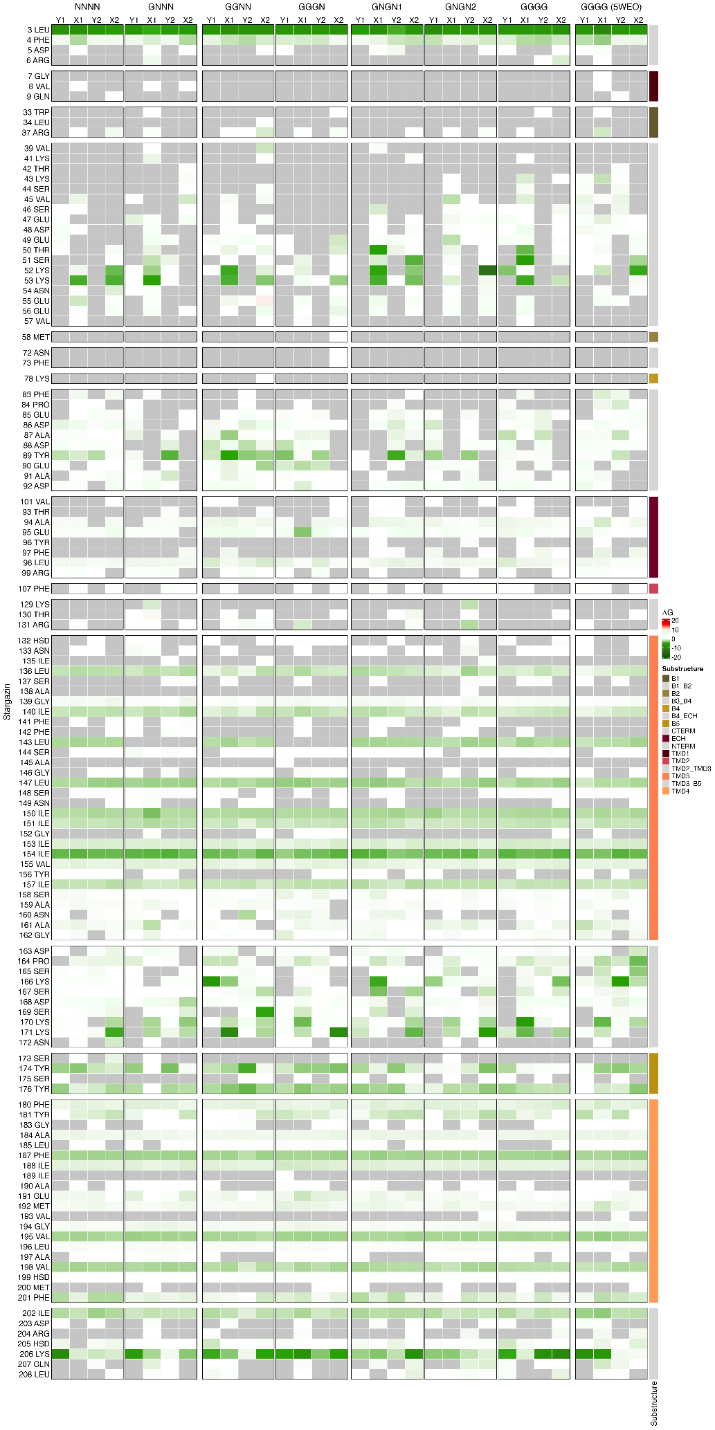


Figure SI 25. ∆G_Binding_ per residue of STG for system with STG V143L. The values are expressed in kcal/mol. The ΔG values were calculated by subtracting the G_AMPAR:STG complex_ G_GluA2_ and G_STG_, which were performed using the AMBER MMPBSA, as implemented in the gmx_MMPBSA package. Residues Ser165, Lys166, and Ser167 (loop between TMD3 and β5) were more stable in the O1 and O2 structures than in the NC structures.


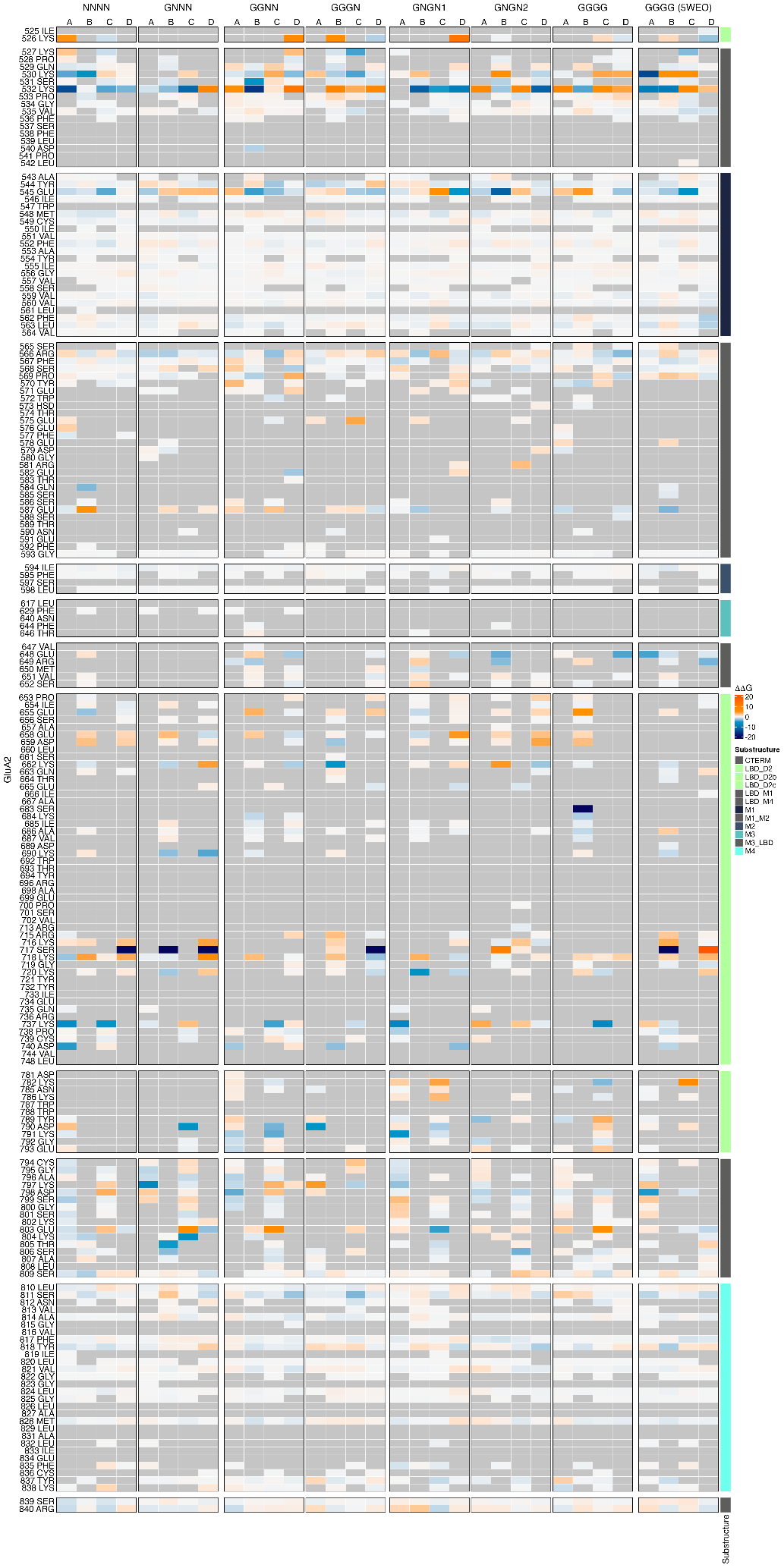


**Figure SI 26. ∆∆G per residue** **for GluA2.** The values were expressed in kcal/mol and were calculated by subtracting the values of ΔG_V143L_ from those of ΔG_WT_. The ΔG values were calculated by subtracting the G_AMPAR:STG complex_ G_GluA2_ and G_STG_, which were performed using the AMBER MMPBSA, as implemented in the gmx_MMPBSA package. The NC structures showed stronger interactions with Lys530 and Lys532 in the STG WT system than in the O2 structure. In chain A of the NNNN system, residues from the LBD_M4 loop (Ser799 to Lys804) were less stable in the mutated system than in the other configurations.


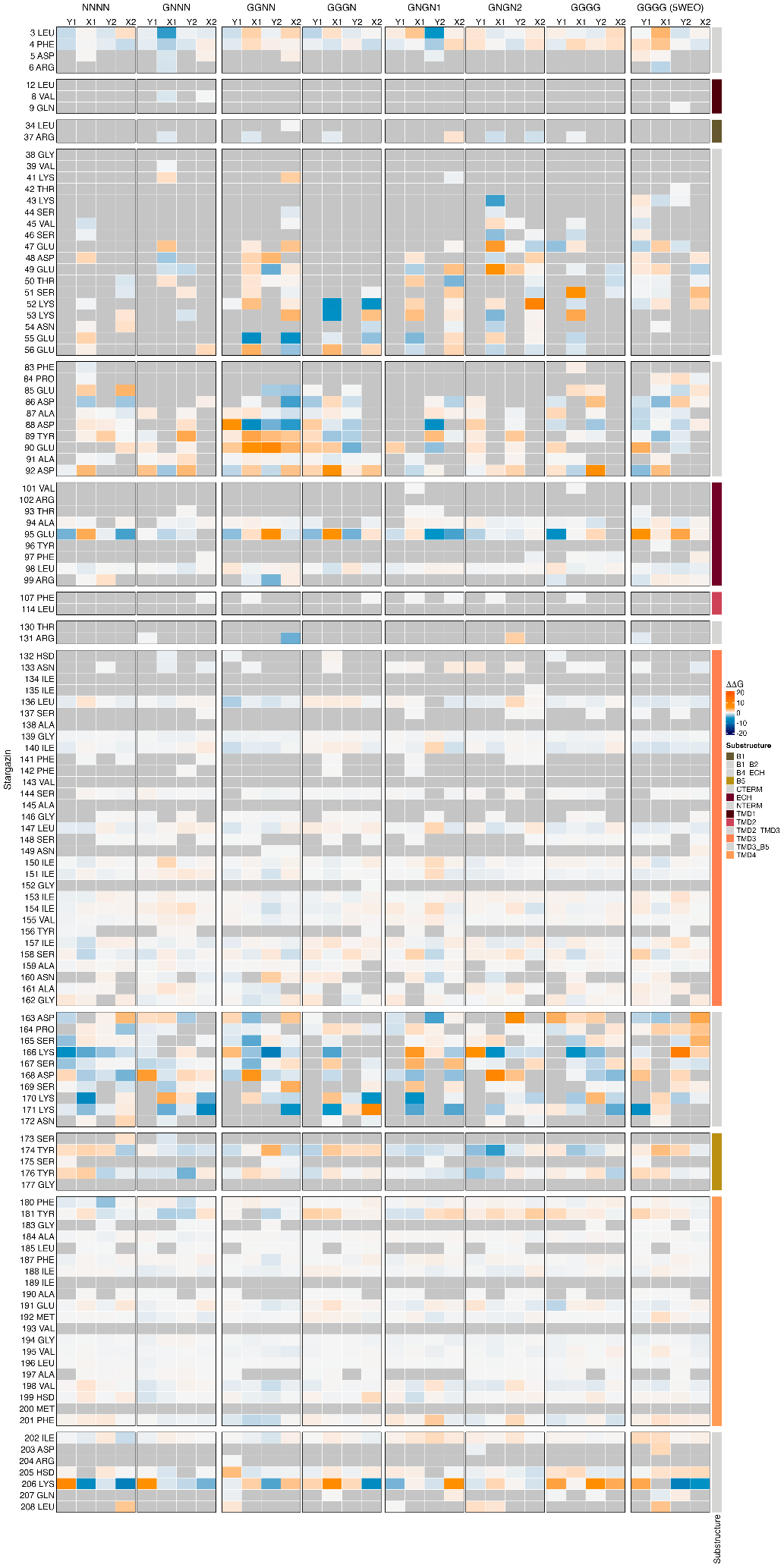


**Figure SI 27. ∆∆G per residue** **for Stargazins.** The values were expressed in kcal/mol and were calculated by subtracting the values of ΔG_V143L_ from those of ΔG_WT_. The ΔG values were calculated by subtracting the G_AMPAR:STG complex_ G_GluA2_ and G_STG_, which were performed using the AMBER MMPBSA, as implemented in the gmx_MMPBSA package. In the presence of the STG V143L mutation, Leu3 and Phe4 (NTERM), Glu95 (ECH), Pro164, Ser165, and Lys166 (loop between TMD3 and β5) were generally more stable in O2 structures, while in NC structures, they were more stable in STG WT systems.
